# Insights into a co-precursor driven solid-state thermal reaction of ferrocene carboxaldehyde leading to hematite nanomaterial: a reaction kinetic study†

**DOI:** 10.1039/d3ra07045j

**Published:** 2023-12-01

**Authors:** Manisha Chakraborty, Sani Kundu, Ashis Bhattacharjee

**Affiliations:** a Department of Physics, Institute of Science, Visva-Bharati University Santiniketan 731235 India ashis.bhattacharjee@visva-bharati.ac.in

## Abstract

Thermal decomposition of a mixture of ferrocene carboxaldehyde and oxalic acid dihydrate in O_2_ atmosphere produced rod-like hematite nanomaterial. The decomposition reaction was complex as evident from the overlapped multistep reaction steps in the non-isothermal thermogravimetry (TG) profiles obtained in the 300–700 K range. A peak deconvolution method was applied to separate the overlapped reaction steps. The multistep TG profiles were successfully deconvoluted, which showed that the decomposition occurs in six individual steps. However, it was found that only the last three reaction steps were responsible for the production of hematite. To estimate the activation energy values for these thermal reactions, six model-free integral isoconversional methods were used. The activation energy value significantly depends on the extent of conversion in each step; however, the nature of its dependence significantly different for each step. The most probable stepwise reaction mechanism functions for the solid-state reactions were obtained using the master plot method. The reaction mechanism was found to be different for different steps. Utilizing the activation energy and reaction mechanism function, the reaction rates of decomposition for each step were determined. To substantiate the validity of the assumed kinetic models, the experimental conversion curves were compared with the constructed ones, and the agreement was quite reasonable. The conversion-dependent thermodynamic parameters were obtained utilising the estimated kinetic parameters. Role of the co-precursor in the thermal reaction of the precursor was plausibly revealed. The present study describes how the use of a co-precursor significantly enhances the thermal decomposition of the precursor, how hematite nanomaterials can be synthesized from a co-precursor driven solid state reaction at low temperatures, and how the kinetic calculations facilitate the understanding of the solid-state reaction process. This study proposes the use of a suitable combination of precursor and co-precursor for solid-state thermal synthesis of iron-based nanoparticles using organo-iron compounds as precursor and also illustrates the effective application of the thermal analysis technique to understand the decomposition reaction.

## Abstract 2

Thermal decomposition of a mixture of ferrocene carboxaldehyde and oxalic acid dihydrate in O_2_ atmosphere produced rod-like hematite nanomaterial. Kinetic analysis of the reaction demonstrated the co-precursor as reaction enhancer.

## Graphical abstract

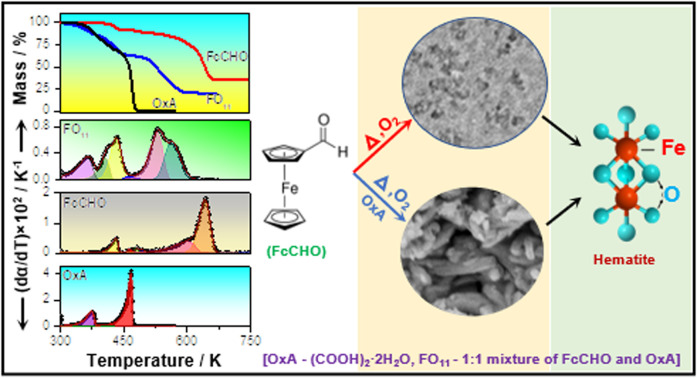

## 1. Introduction

Among several naturally occurring iron oxides, hematite (α-Fe_2_O_3_) possesses the highest thermodynamic stability. Depending on the size and shape, hematite at nanoscale shows a large number of applications.^1–4^ Recent studies demonstrated that fundamental properties of bulk materials can be effectively improved and customized by tuning the size, shape, composition, and crystallinity in nanomaterials.^3–9^ Hematite is an environmentally compatible, cost-effective, and good corrosion-resistive narrow band gap semiconductor. These striking properties led to the fabrication of various nanostructures of hematite (*e.g.*, nanorods, nanotubes, nanocubes, nanospheres, and nanoflakes) by employing a variety of synthesis methods to allow for major applications in magnetic storage devices,^10^ biomedicine,^11^ engineering and industrial fields,^3^ lithium-ion batteries,^12^ gas sensors,^13^ water treatment,^14^ and magnetic resonance imaging (MRI).^15^ However, the physical property of these nanomaterials strongly depend on the size, shape, and crystallinity, which are dependent on the synthesis method.^16^ There are numerous well-known chemical, physical, and biological methods^17^ that have been used to synthesize nanomaterials, among which ∼90% of the chemical methods (*e.g.*, hydrothermal, microemulsion, sonochemical, chemical precipitation, electrochemical, and thermal decomposition)^4,18,19^ are deployed for the preparation of hematite nanoparticles in order to control their size, shape, and crystallinity. Undoubtedly, every method has its merits and demerits. However, among the methods reported so far, the thermal decomposition of organoiron solid precursors is a popular, less expensive technique which is easy to handle.^20–23^ The thermal decomposition of a solid (upon heating a sample) is a process of redistribution of bonds and formation of new products different from the reactants.^24^ This technique has a number of merits such as synthesis at comparatively low temperature, control by reaction time, temperature and environment, faster reaction, and use of a variety of iron-bearing compounds as precursors and organic compounds as co-precursors.^20–23^

Application of kinetic theory of thermal analysis is important to know the underlying process in a solid-state thermal decomposition reaction leading to the formation of new material(s). Among several thermo-analytical methods, the non-isothermal thermogravimetry (TG) is a popular method to explore solid-state thermal reactions,^21,25,26^ where analysis of the TG profiles (*i.e.*, mass *vs.* temperature plots obtained at constant heating rates) based on iso-conversional procedure allows the evaluation of the kinetic parameters (activation energy, reaction mechanism function and reaction rate) and predicts the mechanism of the rate-controlled conversion reaction. The iso-conversional model free integral methods^21,23,25,27–32^ are mostly employed to evaluate the activation energy quite accurately over a range of temperatures utilizing the TG data obtained at different heating rates, and these integral methods are dependent on certain approximations used in solving specific temperature integrals.

Among various organo-iron compounds, ferrocene, (C_5_H_5_)_2_Fe and its derivatives are good sources of iron for the formation of iron oxide nanoparticles through thermal decomposition.^33–37^ Ferrocene decomposes completely at ∼450 K, but produces a hematite nanomaterial when decomposed in presence of oxalic acid.^38^ Acetyl ferrocene,^33^ on decomposition in a nitrogen atmosphere, produced hematite nanoparticles too. Similarly, decomposition of 1-ferrocenyl ethanol in air led to hematite nanoparticles,^21^ while ferrocene carboxaldehyde on thermal decomposition in oxygen atmosphere yielded a much smaller size (av. diameter ∼5 nm) of dot-shaped hematite nanoparticles.^37^ Apart from the reaction environment, additional presence of a co-precursor influences the decomposition process of ferrocene materials, affecting the reaction kinetics.^23,39,40^ For a co-precursor driven thermal decomposition, the co-precursor is chosen in such a way that it decomposes prior to the decomposition of the precursor, so that the decomposed products may react with the intermediate decomposed products from the precursor. In a previous article,^25^ the kinetics of thermal decomposition of ferrocene carboxaldehyde in an oxidative atmosphere was reported.

**Scheme 1:**
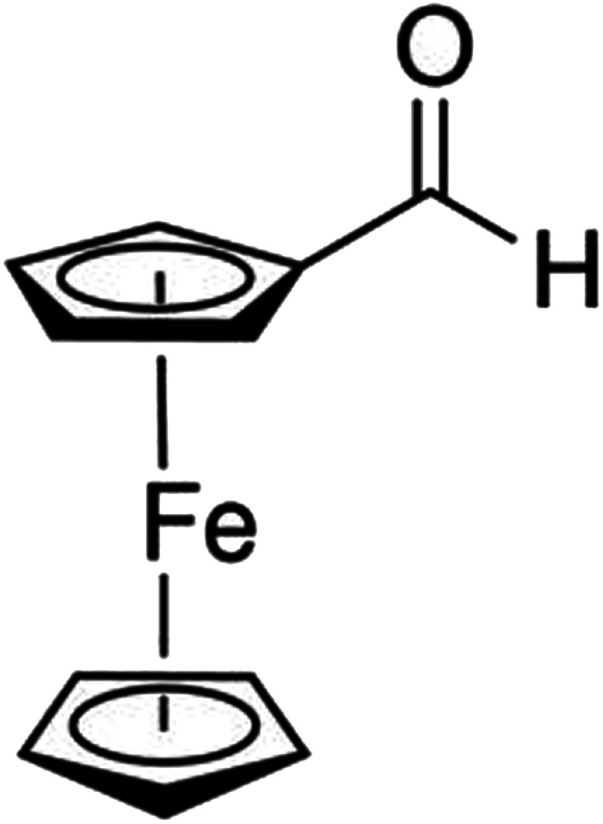
A schematic diagram of ferrocene carboxaldehyde (C_5_H_4_CHO)Fe(C_5_H_5_).

Presently, in order to find the effect of a co-precursor on the nature of solid-state thermal decomposition of ferrocene carboxaldehyde (C_5_H_4_CHO)Fe(C_5_H_5_) (Scheme 1), as well as on the reaction product, we studied the thermal decomposition of this precursor in the presence of oxalic acid dihydrate as a co-precursor in O_2_ atmosphere. Remarkably, oxalic acid dihydrate decomposes to CO_2_, CO, and H_2_O with complete mass loss when heated beyond ∼373 K,^35^ whereas ferrocene carboxaldehyde starts to decompose at ∼380 K.^25^ Here, the TG profiles with multiple heating rates of a 1 : 1 mixture of ferrocene carboxaldehyde and oxalic acid dihydrate were obtained in O_2_ atmosphere, and the decomposed product was identified as a hematite nanomaterial. The multi-step differential thermogravimetry (DTG) profiles were resolved into individual reaction steps by peak-deconvolution technique, and the reaction steps responsible for producing hematite were identified. Model-free non-isothermal methodology was utilized to evaluate the reaction kinetic parameters for each step of the reaction, following the recommendation of the International Confederation of Thermal Analysis and Calorimetry Kinetics Committee (ICTAC).^41,42^ Relevant thermodynamic parameters (Δ*S*, Δ*H* and Δ*G*) were found, and their temperature dependence was studied. A substantial resemblance between the experimentally obtained thermal decomposition profiles and those constructed using the estimated kinetic parameters established the accuracy of the modelling approach applied herein and the methodology adopted. The present work highlights how a co-precursor can affect the decomposition reaction of a precursor to lead to materials of nanometric size. Such results might be extremely useful for researching on the thermal synthesis methodology of nanomaterials of different size, starting with a precursor but by varying the co-precursor.

## 2. Experimental and analytical methods

### 2.1 Material and methods

Ferrocene carboxaldehyde (hereafter, FcCHO) and oxalic acid dihydrate, (COOH)_2_·2H_2_O (hereafter, OxA) were procured from Sigma, and were used as precursor and co-precursor, respectively. Both compounds were mixed in a 1 : 1 weight ratio, and then finely powdered using a mortar pastle. Thermal decomposition of the mixture (hereafter, FO_11_) was performed in a thermogravimetric analyser, TGA (STA 449 F3 Jupiter, Netzsch, Germany) in UHP (99.999%) O_2_ gas environment (flow rate: 50 ml min^−1^) with UHP (99.999%) N_2_ gas as the protective gas (flow rate: 20 ml min^−1^). Heating rate-dependent thermogravimetry (TG) profiles were obtained when the sample of ∼5 mg mass was scanned with an alumina-made sample and reference crucibles.

A powder X-ray diffraction (XRD) study of the decomposed material was performed with a diffractometer D8 Advance of Brucker for the Cu-K_α_ radiation source within the 2*θ* values ranging from 20° to 80°. MATCH (free) software was used for the powder XRD data analysis. Field Effect Scanning Electron Microscope (FE-SEM) images were collected using Gemini SEM 450 of Zeiss, where the Energy Dispersive X-ray Analysis (EDX) study was made with the Model-Z2 of AMETEK. ImageJ software was used for the analysis of the SEM images. For the analysis of the observed thermogravimetric data, the ORIGIN software was used, whereas all calculations for estimation of the reaction kinetic and thermodynamic parameters were done with the help of a program compiled in MATLAB.

#### 2.2.1 Theoretical background of kinetic analysis

In a kinetic study of the thermal solid-state reaction, the rate equation of a single-step thermal decomposition is assumed as,^25^1
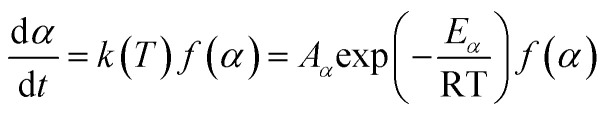
where *α* is the extent of conversion (
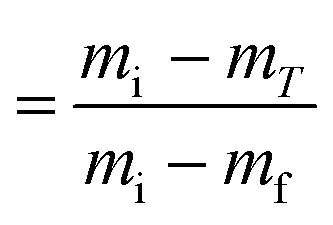
, *m*_i_ is the initial mass, *m*_f_ is the final mass, *m*_*T*_ is the mass at temperature *T*), *k*(*T*) = Arrhenius rate function, *f*(*α*) = conversion function, *A*_*α*_ and *E*_*α*_ are the conversion-dependent reaction rate and activation energy, respectively, while *R* is the universal gas constant. For a non-isothermal reaction process with constant linear heating rate *β*
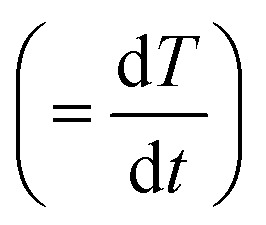
, eqn (1) can be written as,2
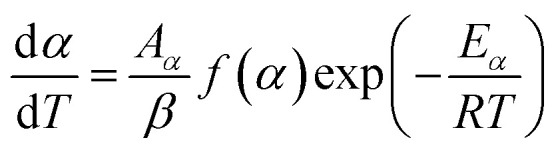


which on rearrangement, reduces to:3
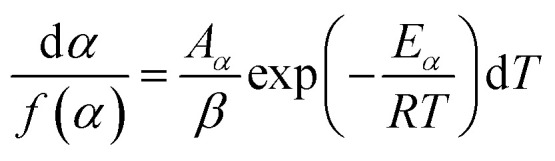


Integrating eqn (3), one obtains the expression for the mechanism function:4
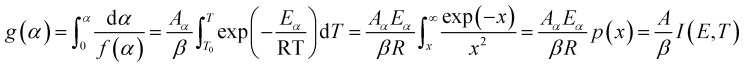
where 
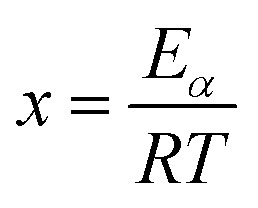
, 
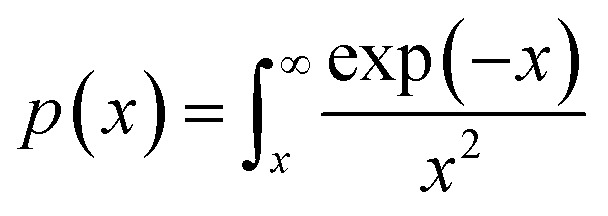
 and integral function 
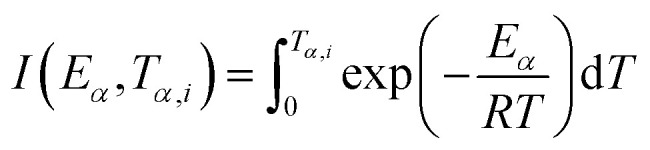
. From eqn (4), it is clearly visible that determination of *g*(*α*) is dependent on the approximation of *p*(*x*). After using the best approximation of *p*(*x*) for a suitable range of *x*, it is possible to calculate the kinetic parameters – *E*_*α*_, *g*(*α*) and *A*_*α*_.

#### 2.2.2 Kinetic parameters

The minimum energy required for the formation of the new bonds in a new complex is known as the activation energy. It is possible to solve the above stated integral *p*(*x*) using modern integral methods utilizing numerical integration and best approximation of *p*(*x*) suitable for a range of *x*,^28–30^ and hence to evaluate the activation energy *E*_*α*_. There are several well-known integral methods, like Flynn–Wall–Ozawa (FWO),^43,44^ Kissinger–Akahira–Sunose (KAS),^45,46^ Tang,^47^ Starink,^29^ Akbi–Mekki–Rafai (AMR),^48^ and Vyazovkin,^28^ defined as:

FWO:^43,44^5a
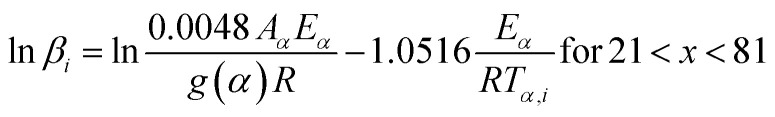


KAS:^45,46^5b
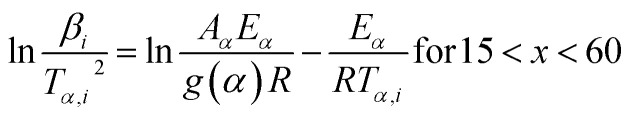


Tang:^47^5c
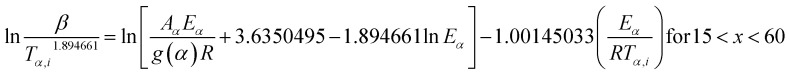


Starink:^29^5d
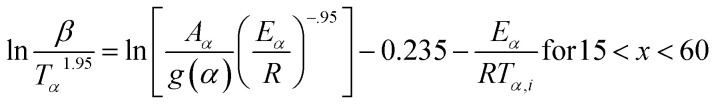


AMR:^48^5e
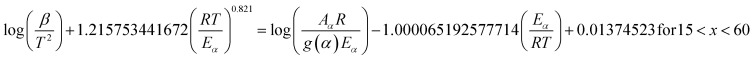


Vyazovkin:^28^5f
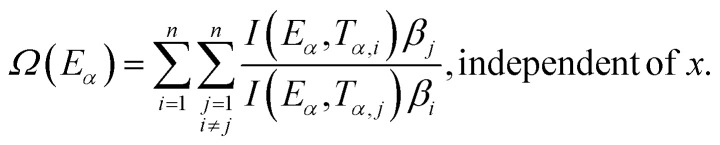


The slope of the linear fit obtained from ln(*β*/*T*^*n*^) *vs.* 1/*T* plots following eqn (5a–d) leads to the estimation of the *E*_*α*_ value. The *E*_*α*_ value can also be derived from eqn (5e) utilizing the method of iteration.^48^ Use of eqn (5f) leads to the estimation of the relatively more accurate value of the activation energy from the minimum value of the objective function *Ω vs. E*_*α*_ plots.^28^ Herein, the equations eqn (5a–f) are applied to evaluate and compare the activation energy values of thermal decomposition.

The mechanism function describes the reaction path followed by the reactants during the product formation, while the reaction rate indicates the frequency of collisions, and hence gives an idea about the reactivity. Different reaction mechanism functions^49^*g*(*α*) are presented in Table S1,† where models like the chemical reaction, random nucleation, phase boundary reaction and diffusion, and subsequent growth are some of the frequently utilized reaction mechanisms. Usually, the master plot method^50^ is used to determine the most probable mechanism function. A master plot is the reference theoretical curve based on the kinetic model function. This method is based on the comparison of theoretical master plots with the experimental master plots. According to eqn (4) with *α* = 0.5 as a reference point, the following equation is obtained:6
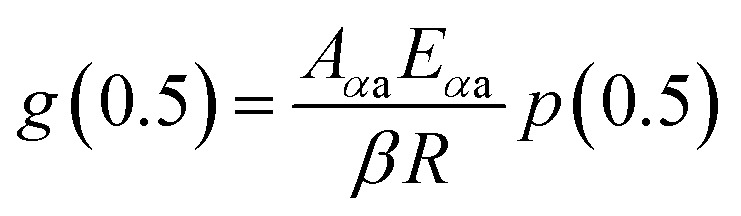
with *A*_*α*a_ = the average value of reaction rate *A*_*α*_ values, *E*_*α*a_ = the average value of activation energy *E*_*α*_ values, *p*(0.5) = *E*_*α*a_/*RT*_0.5_, and *T*_0.5_ is the temperature corresponding to 50% conversion. Dividing eqn (4) by eqn (6), one obtains7
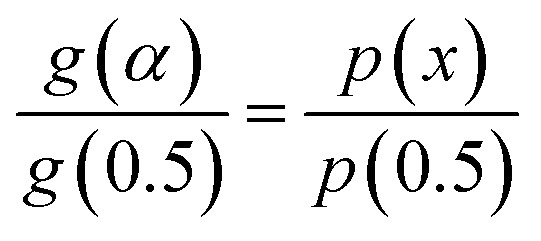


The plot of [*g*(*α*)/*g*(0.5)] as a function of *α* originates the theoretical master plot for a range of *g*(*α*) functions. The experimental master plots are obtained using a suitable approximation of *p*(*x*). When the most probable model is used, eqn (7) shows that for a particular *α*, the experimentally obtained value of *p*(*x*)/*p*(0.5) and the theoretically calculated value of g(*α*)/*g*(0.5) are equal. There are many approximations of *p*(*x*). Among those, the Senum–Young approximation^51^ is a good approximation of *p*(*x*). Using this approximation, *p*(*x*) is written as,8
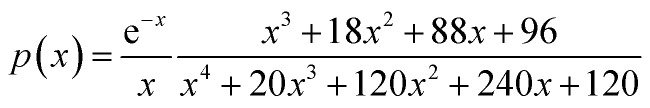


After determining *E*_*α*_ using eqn (5a–f) and *g*(*α*) using eqn (7), the reaction rate *A*_*α*_ is estimated as a function of *α* for different heating rates using the equation:^48^9
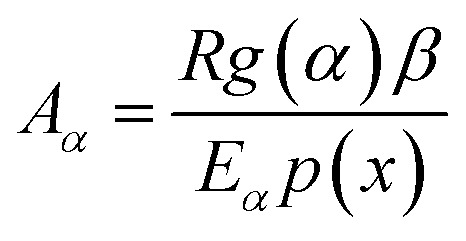


#### 2.2.3 Thermodynamic parameters

The thermodynamic parameters – changes in entropy (Δ*S*), enthalpy (Δ*H*) and Gibbs free energy (Δ*G*) for the activated complex formation during the thermal decomposition can be estimated using the following relations:^21^

10a Δ*S* = *R* ln(*A*_α_*h*/*eχk*_B_*T*_p_)

10b Δ*H* = *E*_*α*_ − *RT*_p_

10c Δ*G* = Δ*H* − *T*_p_Δ*S*

where *e* = 2.7183 (base of natural logarithm); *χ* is the transition factor; *k*_B_ is the Boltzmann constant; *h* is Planck's constant, and *T*_p_ is the peak temperature obtainable from the 
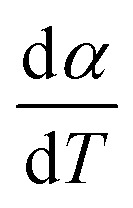
*vs. T* curves.

### 2.3 Peak deconvolution

Eqn (1) represents a single-step decomposition reaction. However, most of the time, thermal decomposition of the iron-based organic solid precursors is a multistep complex reaction process, *i.e.*, they follow multiple consecutive overlapping reaction steps.^21,23,25,52^ For a multistep complex process, the overall rate equation is the combination of multiple single-step reactions.^53^ To separate the individual steps, the peak deconvolution method is a suitable process. For multiple overlapping peaks, the overall 
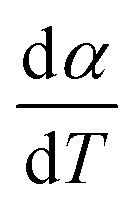
 can be written as11
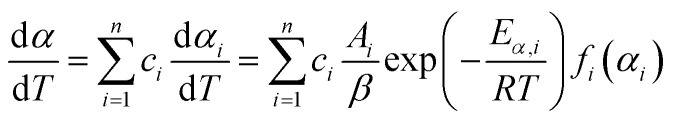
where 
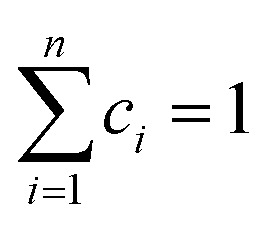
 and 
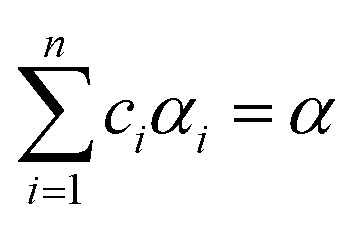
. Here, *n* represents the total number of steps, and *c*_*i*_ denotes the contribution of the *i*^th^ process. *A*_*i*_, *E*_*α*,*i*_ and *f*_*i*_(*α*_*i*_) are the Arrhenius parameters of process *i*, and *α* represents the extent of reaction of the overall process. For a multistep process, the peak deconvolution method is a good approach to separate the overlapping peaks. Perejón *et al.*^54^ used Gaussian, Lorentzian, Weibull and Fraser Suzuki functions to fit 
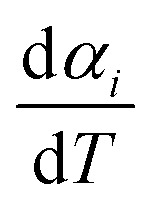
. Considering the asymmetry, the Weibull and Fraser Suzuki functions^21^ were fitted to the experimental 
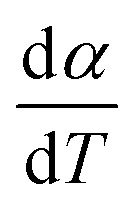
*vs. T* curves. However, in the present study, the fitted curves for the Fraser Suzuki function produced better accuracy than the Weibull function (Fig. S1†). Thus, using the Fraser Suzuki function,^21,23,25^eqn (11) is written as12
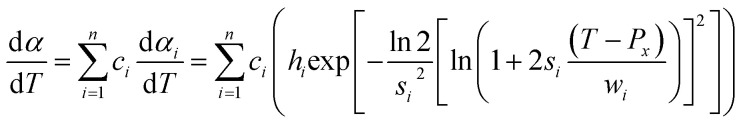
where 
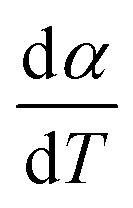
 is the conversion rate of the overall process, and *h*_*i*_, *p*_*i*_, *s*_*i*_ and *w*_*i*_ are the parameters corresponding to the peak amplitude, position, asymmetry and half-width of the *i*^th^ peak, respectively. After setting the initial values of the fit parameters_,_ an optimization operation is done to minimize *F* defined as13
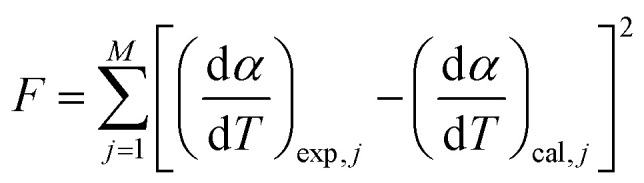
where *M* is the total number of data points under a constant heating rate. The lower value of F provides the better accuracy in fitting. On successful deconvolution, the rate equation for each individual and independent steps of thermal decomposition can be represented by eqn (12) with the derived fit parameters.

## 3. Results and discussion

### 3.1 Analysis of thermogravimetry results

**Fig. 1:**
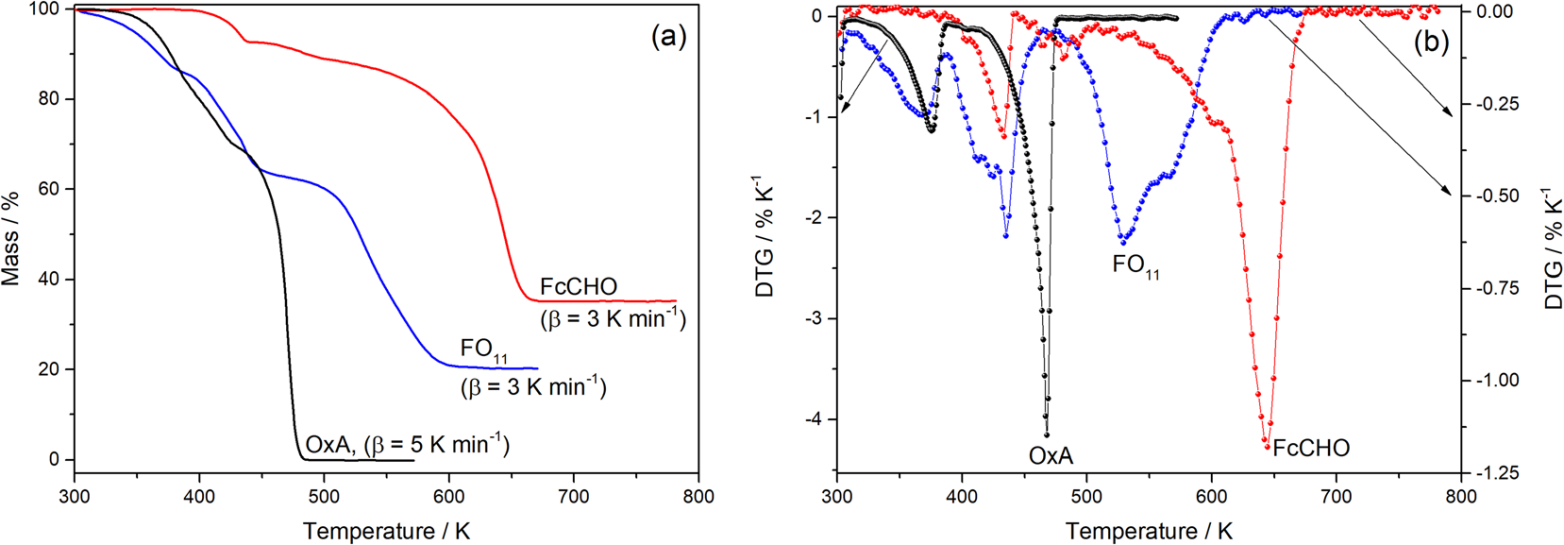
A comparison of the TG (a) and DTG (b) profiles observed for FO_11_, FcCHO, and OxA.

Fig. 1a compares the observed TG profile of FO_11_ in an O_2_ atmosphere with those obtained for its components, OxA and FcCHO. The TG profile of OxA obtained under 5 K min^−1^ heating rate shows that it starts to decompose at ∼320 K and decomposed completely at ∼474 K following a three-step process, while the TG profile of FcCHO obtained under 3 K min^−1^ heating rate shows that decomposition started at ∼380 K and was completed at ∼678 K with 35% of residual mass, seemingly following a three-step process. On the other hand, thermal decomposition of FO_11_ starts at ∼305 K and is completed at ∼625 K with 20% residual mass, exhibiting an apparent four-step process. The mass loss associated with each decomposition step in the TG profiles is due to the release of volatile material(s), owing to the progress of the solid-state decomposition reaction. With the progress of reaction, the reaction steps occur at different temperatures, and the reaction is completed when a thermally stable compound is obtained. Comparing the TG profiles, as FcCHO is seen to be stable up to ∼380 K from the TG profile, the initial mass loss of FO_11_ observed in the 305–380 K range should be due to OxA decomposition. After OxA decomposes completely (at ∼474 K), any mass loss in FO_11_ should be due to decomposition of FcCHO. Hence, as FO_11_ is a physical mixture of FcCHO and OxA, one can expect that the TG profile of FO_11_ should be similar to that of FcCHO above ∼474 K. However, this is not the result observed, as reflected in Fig. 1a. The nature of TG profile of FO_11_ is quite different from that of FcCHO (shifted towards lower temperature compared to FcCHO) and the decomposition of FO_11_ is completed at ∼625 K, which is about 50 K lower than that of FcCHO.

Fig. 1b shows the differential thermogravimetry (DTG) profiles obtained from the TG data for OxA, FcCHO and FO_11_ shown in Fig. 1a, where the existence of multiple peaks/humps in a single profile indicates the occurrence of multi-step thermal decomposition reactions and the positions of the peaks/humps are the temperatures where maximum decomposition takes place in a particular reaction step. Here, OxA shows two distinct peaks at 375 K and 467 K, along with a hump at ∼404 K. Thermal decomposition of FcCHO shows three peaks, along with small humps at 433 K, 465 K (hump), 482 K (hump), 493 K (hump), 601 K and 644 K, in agreement with a previous report.^25^ Apparently, the DTG profile of FO_11_ shows three large peaks at 364 K, 433 K and 529 K, along with shoulder peaks/humps at 408 K, 465 K and 564 K. Thus, a comparison of the DTG profiles of FO_11_ and its constituents shows that there is a clear shift in the observed DTG peak positions when FcCHO is decomposed in the presence of OxA. Hence, the observations made from the analysis of the TG and DTG profiles of OxA, FcCHO and FO_11_ (shown in Fig. 1) undoubtedly establish that the presence of OxA in the solid reaction atmosphere significantly influences the thermal decomposition of FcCHO.

**Fig. 2:**
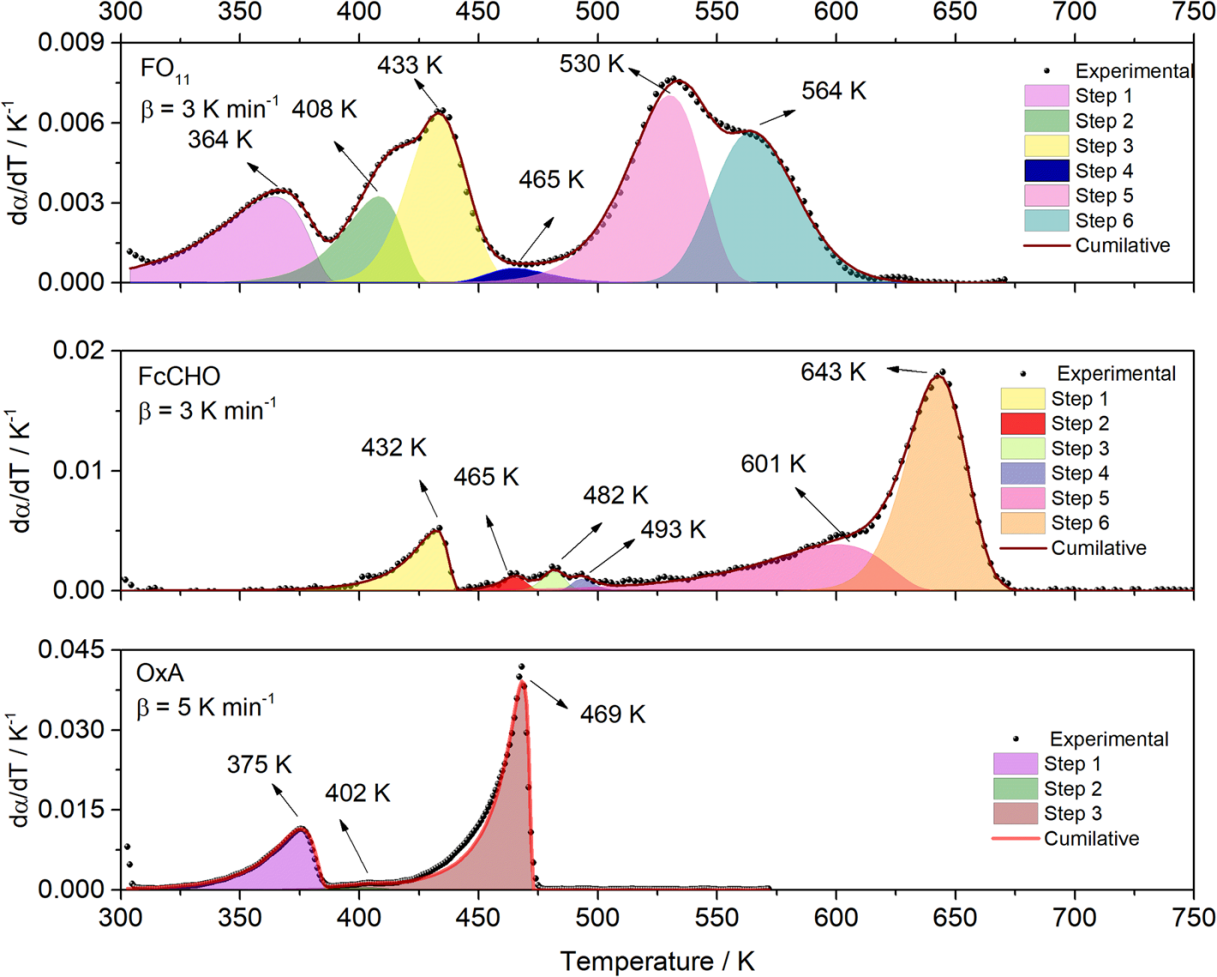
The deconvoluted 
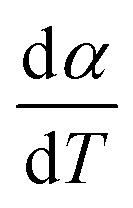
*vs. T* profiles of FO_11_, FcCHO, and OxA.

Fig. 1 showed that thermal decomposition of FO_11_ in an O_2_ atmosphere is a complex multistep process. Hence, the peak deconvolution method following the Fraser Suzuki function, as discussed earlier, was adopted to separate the overlapping DTG peaks of FO_11_, FcCHO and OxA. The fit parameters are available as supplementary material (Table S2†). Fig. 2 compares the deconvoluted 
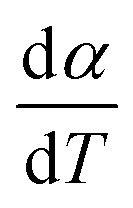
*vs. T* plots for FO_11_, FcCHO and OxA with the experimental ones for a single heating rate, where the agreement is very good. The peak deconvolution method shows that thermal decomposition of FO_11_ takes place in six different steps, where the steps are quite overlapped, while OxA and FcCHO decompose in three and six different steps, respectively, in agreement with earlier reports.^23,25^Table 1 compares the temperature corresponding to the maximum decomposition (*T*_p_) for each reaction step and the temperature range of steps (Δ*T*) for the decomposition of FO_11_, FcCHO and OxA in an O_2_ atmosphere. From Fig. 2 and Table 1, when the reaction steps of individual FcCHO and OxA are compared with those of FO_11_, it is imminent that peak-1 and peak-2 observed for FO_11_ are due to the evaporation of adsorbed water molecules, followed by partial decomposition of OxA, and peak-3 is due to the initial decomposition of FcCHO. OxA starts to decompose much before FcCHO, and Step 1 of OxA completes at ∼387 K, while FcCHO starts its Step 1 of decomposition at around the same temperature. Peak-4 observed for FO_11_ is low in intensity. Notably, peak-3 of OxA and peak-2 of FcCHO appear at about the same temperature at lower heating rates. Eventually, peak-2, 3, 4 of lone FcCHO may be present in the same temperature range. However, considering the level of error, any further attempt to deconvolute the low intensity curve (*i.e.*, peak-4 for FO_11_) was avoided. Heating rate-dependent 
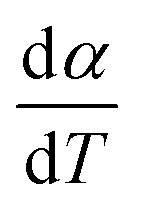
*vs. T* plots of OxA (see Fig. S2†) show that the intensity of peak-2 increases with increasing heating rate, and peak-3 is sustained at higher heating rates when lone OxA is heated in an O_2_ atmosphere. This discards the possible merger of peak-3 of OxA with peak-2 of FcCHO as peak-4 in FO_11_. The disappearance of peak-3 of OxA in FO_11_ is inquisitive. On the other hand, with increasing heating rate, peak-2 of lone FcCHO decreases faster in intensity and shifts towards higher temperatures.^25^ It will be shown in the later discussions that with increasing heating rate, peak-4 observed for FO_11_ gradually decreased in intensity and shifted towards higher temperature. The positions of *T*_p_ for Step 5 and -6 for FO_11_ are significantly different than those of lone FcCHO. Thus, it may be presumed that in FO_11_ under O_2_ atmosphere, after the Step 2 decomposition of OxA, the gaseous products that are formed react with those formed by Step 1 decomposition of FcCHO with increasing temperature, leading to Step 4, -5 and -6 of reactions in FO_11_, as well as the residual product. This causes the different TG and DTG profiles for thermal decomposition of FO_11_ in comparison to that of FcCHO.

**Table 1:** Values of the temperature corresponding to maximum decomposition (*T*_p_) for each reaction step and the temperature range of steps (Δ*T*) for decomposition of FO_11_, ferrocene carboxaldehyde, and oxalic acid in O_2_ atmosphere

Material	Parameter	Step 1	Step 2	Step 3	Step 4	Step 5	Step 6
FO_11_ @ 3^a^ K min^−1^	*T*_p_ (K)	364 K	408 K	433 K	465 K	530 K	564 K
	Δ*T* (K)	300–390 K	332–432 K	382–468 K	439–515 K	440–568 K	515–625 K
FcCHO @ 3^a^ K min^−1^	*T*_p_ (K)	432 K	465 K	482 K	493 K	601 K	643 K
	Δ*T* (K)	380–440 K	446–479 K	464–494 K	472–512 K	452–641 K	585–678 K
OxA @ 5^a^ K min^−1^	*T*_p_ (K)	375 K	402 K	469 K			
	Δ*T* (K)	304–387 K	378–414 K	380–474 K			

a Heating rate.

### 3.2 Identification of the decomposed material

**Fig. 3:**
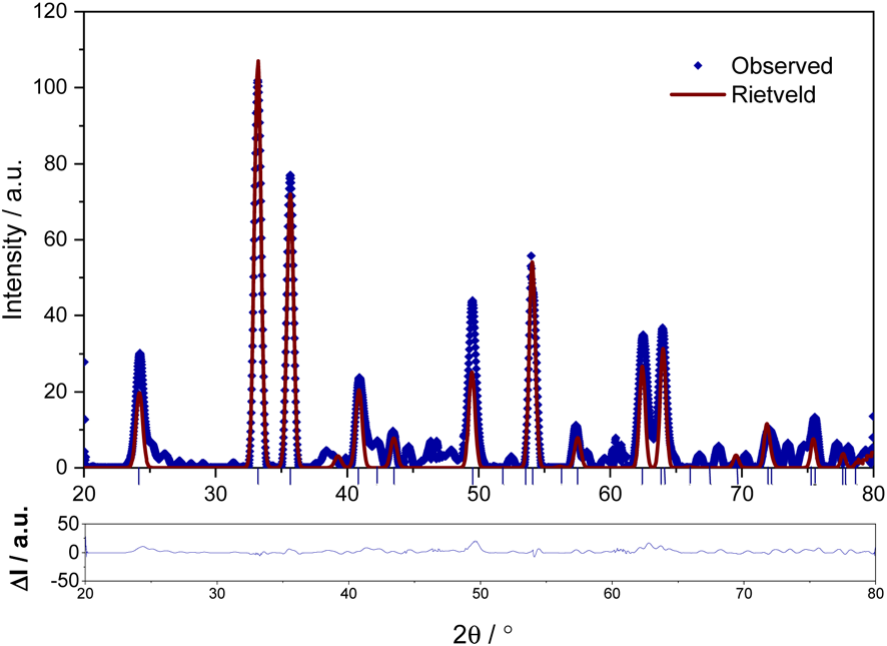
The powder XRD profile of the decomposed material obtained on thermal decomposition of FO_11_ in O_2_ atmosphere. The lower panel shows the difference between the observed and fitted XRD profiles.

In order to identify the residual product obtained on thermal decomposition of FO_11_ in O_2_ atmosphere, a room temperature powder XRD pattern was obtained in the 2*θ* range of 20°–80°. Fig. 3 shows the observed powder XRD pattern along with the Rietveld refinement results, where the dot represents the observed data, the solid line corresponds to the calculated pattern, and the vertical lines indicate the Bragg diffraction positions. Clear distinct Bragg peaks are observed at 24.20°, 33.28°, 35.69°, 40.89°, 49.61°, 54.17°, 62.52° and 63.99°. Close agreement between the observed and calculated powder XRD patterns recognized that the thermally decomposed material obtained from FO_11_ in the O_2_ atmosphere is pure hematite (α-Fe_2_O_3_) with a hexagonal crystal structure with *a* = *b* = 5.0428 Å, *c* = 13.7326 Å and *α* = *γ* = 90° and *β* = 120^°^ (COD No: 96-901-5066).^55^ The sharp and intense peaks of α-Fe_2_O_3_ imply the absence of impurities and good crystalline nature of the sample.

The average particle size of the hematite material derived using the Scherrer method^56^ and Wagner–Halder method^57^ is 12.51 ± 0.02 nm and 10.72 ± 0.03 nm, respectively. So, the average particle size values estimated using both methods are consistent with each other and lie in the nanometric range. Thus, it is recognized that on thermal decomposition of ferrocene carboxaldehyde in the presence of oxalic acid dihydrate in an oxidative atmosphere completed at 625 K, pure hematite nanoparticles can be obtained. At this point, it may be recalled that the thermal decomposition of lone ferrocene carboxaldehyde completed at ∼678 K in oxygen atmosphere produced dot-shaped hematite nanomaterials of ∼5 nm av. diameter.^37^

**Fig. 4:**
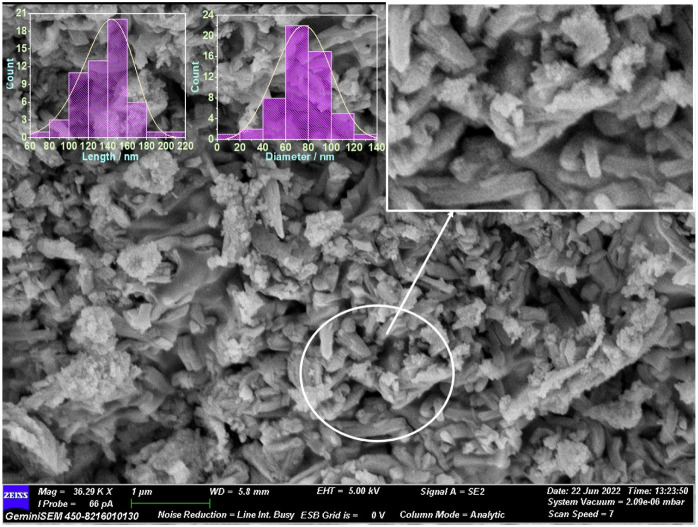
FE-SEM image of the decomposed material of FO_11_. The inserts show the magnified image and histograms.

FE-SEM image was used to analyse the morphology of the prepared hematite sample (Fig. 4). The images revealed that the hematite material has rod-like forms, interestingly different from the spherical nanoparticles obtained when bare FcCHO was decomposed under the same oxidative atmosphere. Some nanorods were randomly selected and then processed with ImageJ software to obtain the size distribution of the resulting particles (shown as insert of Fig. 4). The average length and diameter of the rods obtained from the histogram are 148 ± 6 nm and 83 ± 3 nm, respectively. Next, EDX analysis was used to measure the proportion of elemental species contributing to the synthesized hematite nanorods. The observed EDX spectrum is shown in Fig. S3,† which verified the presence of 57.0% of iron and 43.0% of oxygen (atomic weight percentage) species, confirming that the thermally synthesized hematite is made up of only iron and oxygen with no impurities.

### 3.3 Estimation of kinetic parameters

**Fig. 5:**
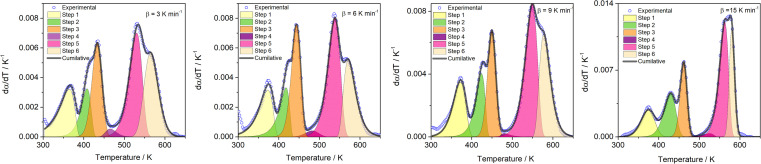
The results of the peak deconvolution analysis (
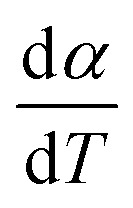
*vs. T* plots) for each separate step of the thermal decomposition of FO_11_ at different heating rates under O_2_ atmosphere.

Here, we try to understand the reaction processes involved by estimating the reaction kinetic parameters. The TG profiles of FO_11_ under O_2_ atmosphere at different heating rates were obtained, where the DTG (see Fig. S4†) showed multiple overlapping peaks/humps. The peak-deconvolution method, as discussed earlier, is applied to separate the individual peaks/humps, and the results obtained are shown in Fig. 5. The fit parameters are available as supplementary material in Table S3.† In this table, the different values of *w* for different heating rates indicate the different reactivities for the separated reactions. The different values of *h* with each heating rate signify the different intensities. A positive value of *s* signifies the reaction is right symmetric, while a negative value of *s* signifies the reaction is left symmetric.^58^Fig. 5 compares the deconvoluted 
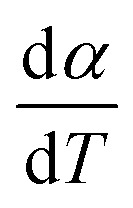
*vs. T* plots for FO_11_ with the experimental ones obtained from the TG data at different heating rates, where the agreement between the calculated (cumulative) and the experimentally observed 
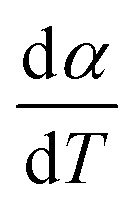
*vs. T* plots is very good. The observed shift in *T*_p_ towards the higher temperature with increasing heating rate may be due to the lowered heat transfer rate between the furnace of TGA and the sample. From the estimated Δ*T* and *T*_p_ values (Table S3†), thermal decomposition of FO_11_ in O_2_ occurs in six overlapped steps in the 300–419 K, 332–465 K, 382–488 K, 439–545 K, 440–586 K and 515–635 K temperature ranges having *T*_p_ values (averaged over heating rates) equal to ∼371 K, ∼420 K, ∼447 K, ∼488 K, ∼545 K and ∼573 K, respectively. Thus, as stated above, the first three peaks (*i.e.*, first three steps) that appeared for FO_11_ are due to decomposition of FcCHO and OxA in the O_2_ atmosphere, whereas the last three peaks (*i.e.*, last three steps) are for the thermal reactions leading to hematite. Considering the aim of the present study, we will focus only on the last three reaction steps.

**Fig. 6:**
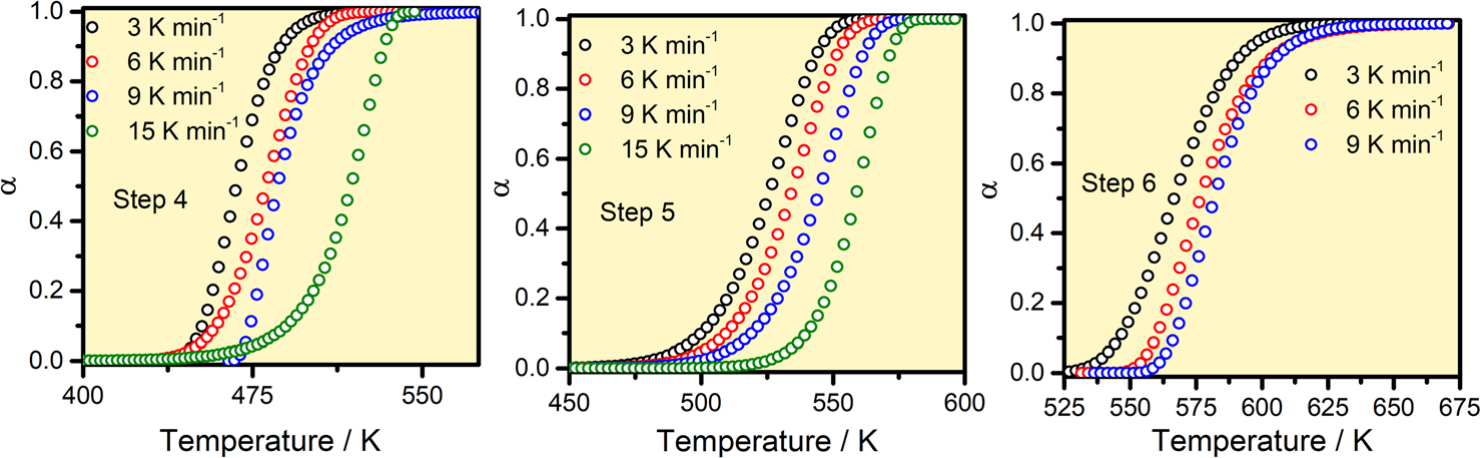
The *α*–*T* curves for Steps 4, 5, and 6 for the thermal decomposition of FO_11_.

For kinetics analysis, the extent of conversion *α* for each step of decomposition of FO_11_ was calculated as a function of temperature using the relation 
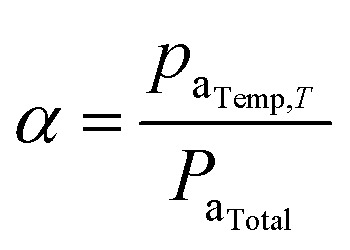
,^25^ where *p*_a_Temp,*T*__ and *p*_a_Total__ are the area of the d*α*/d*T vs. T* curve up to a temperature *T* and the area under the individual *d*α/d*T vs. T* curve, respectively for a particular heating rate. The *α vs. T* plots thus obtained for Step 4, -5 and -6 of FO_11_ are shown in Fig. 6.

**Fig. 7:**
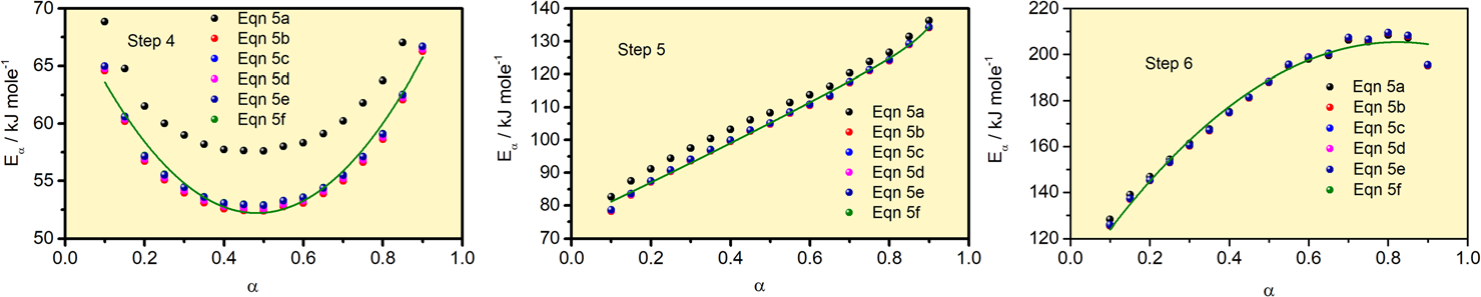
*E*_α_ *vs. α* curves for Step 4, 5, and 6 of FO_11_ using different methods eqn (5a–f). Solid lines are the fits to the estimated values.

The *E*_*α*_ values for 0.1 ≤ *α* ≤ 0.9 for the thermal decomposition of FO_11_ for Step 4, -5 and -6 are estimated by the FWO, KAS, Tang, Starink, AMR and Vyazovkin methods using eqn (5a–f). The ln(*β*/*T*^*n*^) *vs.* 1/*T* curves obtained for Step 4, -5 and -6 are compared in Fig. S5,† while Fig. S6† shows the *Ω*(*E*_*α*_) curves according to eqn (5f) for the same steps. Table S4† presents the results of linear fitting of the experimental data with eqn (5a–f). The *E*_*α*_ values thus estimated using eqn (5a–f) are presented in Table S5.†Fig. 7 shows the variations of these *E*_*α*_ values for thermal decomposition of FO_11_ for Step 4, Step 5 and Step 6 with the extent of conversion *α* in an O_2_ atmosphere. For step 4, the *E*_*α*_ values estimated using the FWO method lie between 57 and 71 kJ mol^−1^, whereas for the rest of the methods, the *E*_*α*_ values lie between 52 and 67 kJ mol^−1^. Meanwhile, for Step 5 and Step 6, the *E*_α_ values estimated following different methods are quite close and lie within 82–137 kJ mol^−1^ and 128–207 kJ mol^−1^ for Step 5 and -6, respectively. As the *E*_*α*_ values for each reaction step using eqn (5a–f) are quite close to each other and particularly, those estimated using eqn (5b–f) are the same, the *E*_*α*_ values estimated using eqn (5f) will be preferentially used for further calculations of the kinetic parameters.

The nature of dependence of *E*_*α*_ values on *α* obtained following different equations are identical for each individual step. The *E*_*α*_ values significantly depend on the extent of conversion in each step. However, the nature of dependence of *E*_*α*_ on *α* is significantly different from step to step. The *E*_*α*_ values are estimated for some distinct *α*. Since *E*_*α*_ is continuous, we interpolate *E*_*α*_ values for all *α* by fitting with suitable functions. In Step 4, the *E*_*α*_ value initially decreases up to *α* = 0.5, and then increases following a polynomial equation of second order. In Step 5, the *E*_*α*_ value increases with an increase in *α*, following an exponential growth equation. Meanwhile, in Step 6, the *E*_*α*_ value increases with an increase in *α*, following a polynomial equation of second order. The functions are available in Table S6.† Since the activation energy is the energy barrier to overcome for a reaction process to occur, the higher values of *E*_*α*_ with the increase of reaction step (*i.e.*, temperature) indicate that more energy is required for the formation of the corresponding products with increasing temperature. In other words, it can be stated that the higher activation energy indicates the high thermal stability of the activated complex. An increasing trend of the activation energy with increasing temperature indicates competing, independent or consecutive reactions.^59^ Generally, this type of reaction occurs during a solid to (solid + gas) transformation. Usually, the gas–solid reaction is a heterogenous reaction existing with multiple parallel reactions. If the dominant reaction path or the reaction control steps change, the activation energy may vary significantly.^60^ The decreasing trend of the activation energy with increasing temperature indicates an endothermic reversible reaction, followed by an irreversible reaction.^61^

**Fig. 8:**
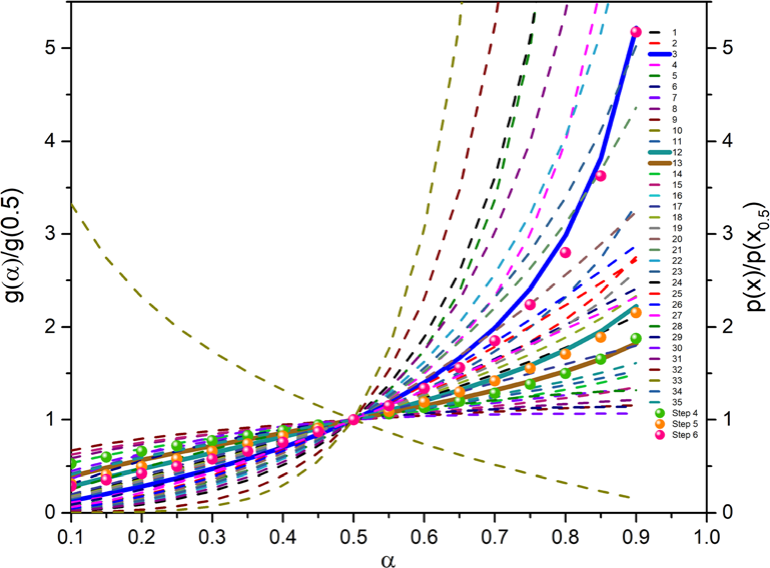
The master plot curves of FO_11_ for Step 4, 5, and 6.

The most probable reaction mechanism functions *g*(*α*) for the present solid-state reactions are obtained following the master plot method described earlier using all 35 mechanisms (shown in Table S1†). Fig. 8 shows the comparison of the theoretical and experimental master plots for FO_11_ in Step 4, 5 and 6. From Fig. 8, it is clear that none of the experimental plots exactly coincide with the theoretical master plot. Hence, to determine the most probable reaction mechanism function, the standard deviation between the theoretical master data and experimental data^50,52^ was calculated, and then the function for which the *Σδ* became minimum was chosen as the most probable reaction function. The expression for *Σδ*^52^ is stated as14
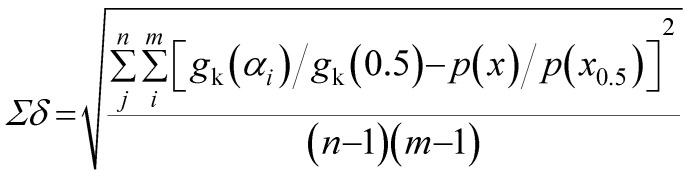
where *m* = number of data points and *n* = number of heating rates, and *δ* = averaged square value of the deviation between *p*(*x*)/*p*(*x*_0.5_) calculated from the experimental data and *g*_k_(*α*_*i*_)/*g*_k_(0.5) calculated for any theoretical model.

The most probable mechanism functions for thermal decomposition of FO_11_ (described in Table S7†) are the sigmoidal reaction (*A*_2_), sigmoidal reaction (*A*_3/2_) and chemical reaction (*F*_3/2_) for Step 4, -5 and -6, respectively. So, the thermal decomposition of FO_11_ in an O_2_ atmosphere follows different reaction mechanisms for different steps. The chemical reaction represents the bond redistribution between the reactants and products. The acceleratory rate equations indicate nucleation without considering any restriction of nuclear growth. For these kinds of reactions, the rate of the reaction increases with the conversion. Sigmoidal equations represent the random nucleation and growth of new nuclei. In the nucleation process, two types of chemical change may be involved – (i) the chemical transformation of one or more constituents of the reactant into the constituents of the product, and (ii) recrystallization of the reacted material to the lattice structure of the product phase. The effect of nucleation causes the active reactant/product interface generation. The reaction occurs in these interfaces during its subsequent advance into an unchanged reactant as the nucleus grows. Growth is thus the maintained reaction within the interface between the reactant and product phases. For such reactions, the nucleation first occurs at local nucleation sites, and then the nucleation sites are eliminated through the growth of new nuclei.^62^ Hence, the rate of the reaction first increases and then decreases with the conversion of the reaction.

**Fig. 9:**
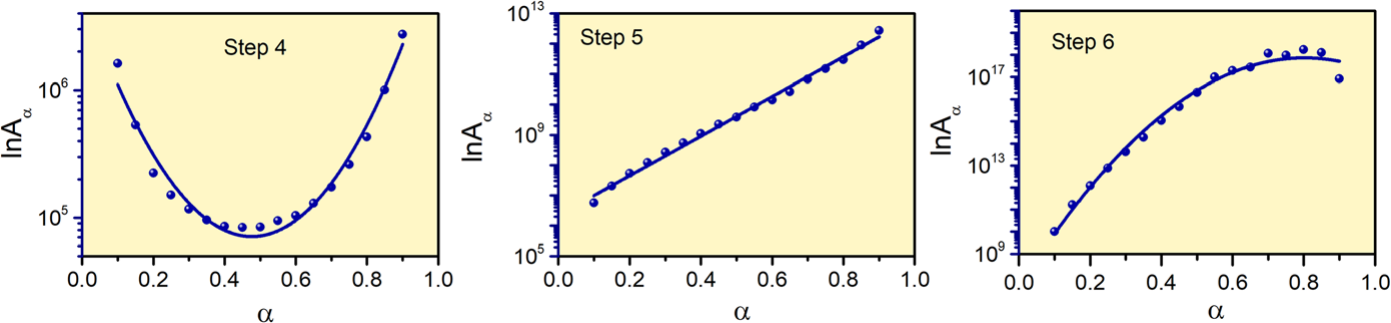
The variations of the reaction rate with the extent of conversion for Step 4, 5, and 6 of FO_11_. Solid lines are the fits to the estimated values.

The reaction rate values of Step 4, -5 and -6 for thermal decomposition of FO_11_ as a function of *α* are calculated from the estimated *E*_*α*_ and *g*(*α*) using eqn (9), and are presented in Table S8.†Fig. 9 shows the variations of ln*A*_α_ with *α* for Step 4, -5 and -6 of FO_11_. The *α*-dependent *A*_*α*_ values of for Step 4, -5 and -6 lie within the range 8.4 × 10^4^–2.7 × 10^6^ min^−1^, 5.6 × 10^6^–2.7 × 10^12^ min^−1^ and 9.9 × 10^9^–1.8 × 10^18^ min^−1^, respectively. Reaction rate *A*_α_ represents the frequency of the collision in the activated complex. For surface-independent reactions, the higher values of *A*_*α*_ indicate a ‘loose’ complex (>10^9^ s^−1^) indicate a highly reactive system, while a low value of *A*_*α*_ indicates a ‘tight’ complex and obviously a less reactive system.^63^ The high values of the reaction rate for Step 6 indicates the high reactivity of the activated complex. The increasing trend of *A*_*α*_ with increasing temperature indicates that the reactivity of the activated complex increases with the extent of conversion. The nature of variation of ln*A*_*α*_ with *α* for each step is identical with the corresponding *E*_*α*_*vs. α* plots.

### 3.4 Reliability of the kinetic models used

When the estimated kinetic parameters are combined, the observed step-wise solid-state thermal decomposition of FO_11_ in an O_2_ atmosphere can be summarised as follows:

Step 4: 15a
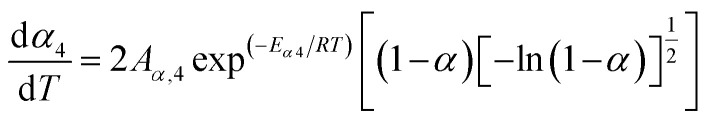


Step 5: 15b
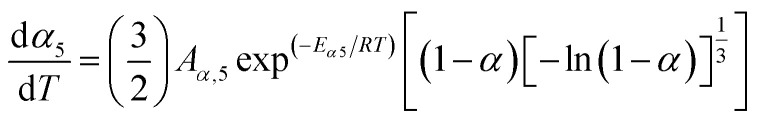


Step 6: 15c
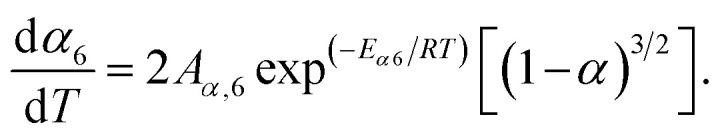


**Fig. 10:**
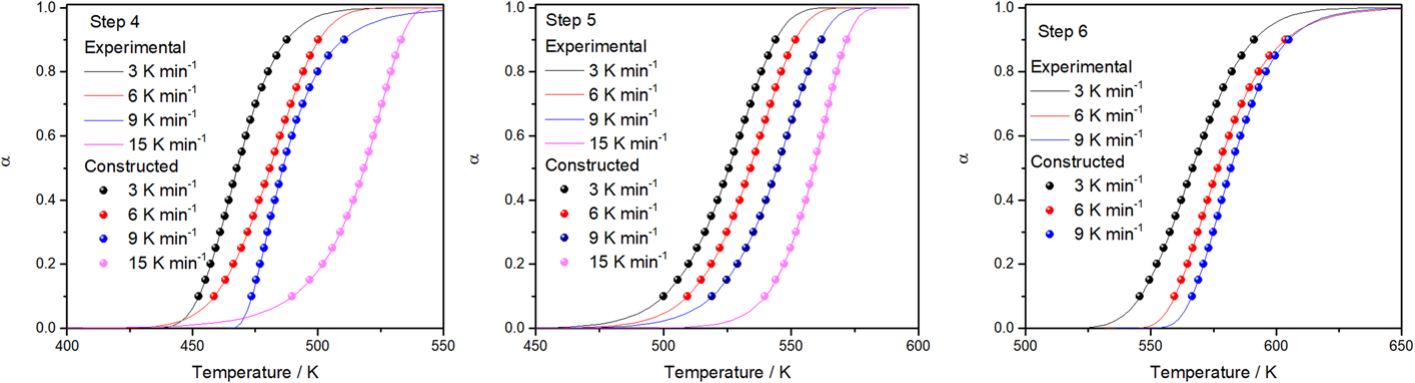
A comparison of *α vs. T* plots for Step 4, 5, and 6 obtained using the kinetic parameters and experimentally observed for thermal decomposition of FO_11_ at different heating rates.

To verify the reliability of the used kinetic models as well as the validity of the estimated reaction kinetic parameters for the thermal decomposition of FO_11_ leading to hematite nanoparticles, the conversion curves (*α vs. T* plots) under different heating rates are made following eqn (14), and are then compared with those obtained from experimental data. Fig. 10 shows such constructed *α vs. T* plots for different heating rates with the corresponding experimentally obtained ones. There is a reasonable agreement between these *α vs. T* plots, which specifies the precision and utility of the methodology implemented for the kinetic analysis and the choice of appropriate models for different steps of reaction in the present study.

### 3.5 Estimation of thermodynamic parameters

**Fig. 11:**
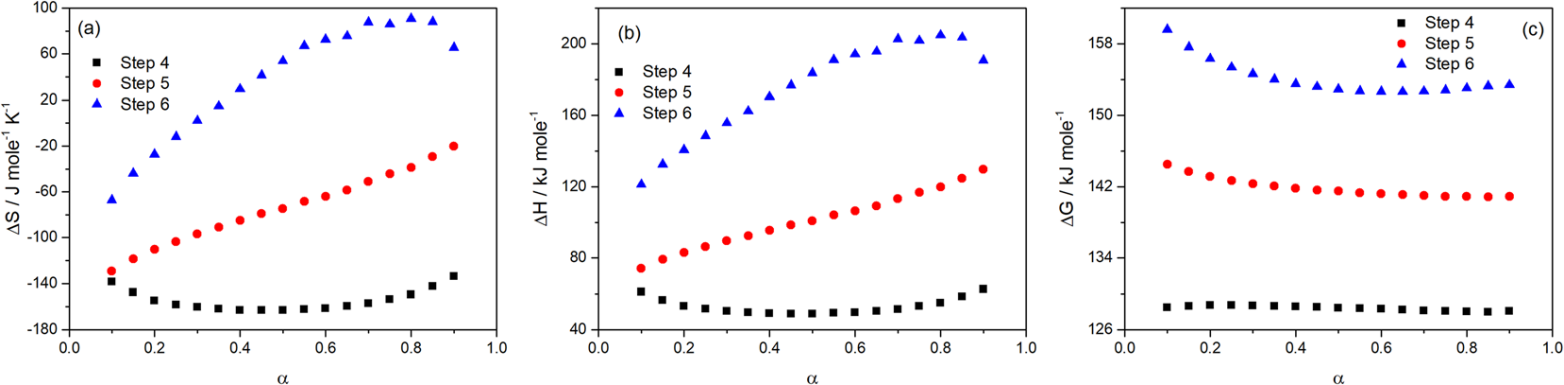
Variations of Δ*S* (a), Δ*H* (b) and Δ*G* (c) with the extent of conversion for Step 4, 5 and 6 of FO_11_.

The values of the thermodynamic parameters Δ*S*, Δ*H*, and Δ*G* for Step 4, 5, and 6 of thermal decomposition of FO_11_ in O_2_ atmosphere are calculated using eqn (10a–c) using the estimated kinetic parameters. The estimated values are found to be heating rate-independent. Thus, their values averaged for different heating rates were considered for discussion. The values of Δ*S*, Δ*H* and Δ*G* are presented in Table S9,† and are plotted in Fig. 11 as a function of *α*.

From Fig. 11a, for Step 4, the Δ*S* values decrease with increasing *α* up to *α* = 0.5, followed by an increase up to *α* = 0.9 and lie within −138.5 to −134 kJ mol^−1^ K^−1^. For Step 5, the Δ*S* values linearly increase with increasing *α* and lie within −129.1 to −20.2 kJ mol^−1^ K^−1^. For Step 6, the Δ*S* values increase with increasing *α* up to *α* = 0.8, followed by a decrease up to *α* = 0.9 and lie within the range of −67.3 to –90.7 kJ mol^−1^ K^−1^. Δ*S* implies the change in the entropy values while going from the intermediate reactant state to the transition state, and provides a measure of the degree of randomness in the system. A negative value of Δ*S* corresponds to a low degree of freedom in the activated complex and *vice versa*. Consequently, these can be referred to as the “slow” and “fast” stages of the reaction, respectively. A lower entropy value implies that the substance is closer to its equilibrium state. Consequently, it will be less reactive and a longer time is required to form the activated complex. Equally, a higher entropy value implies that the system is far-off from its equilibrium. Thus, it is highly reactive and will not take a longer time to form an activated complex.

The energy required by a system for the reaction to occur is the enthalpy. The difference in the potential energy between the activated complex and the precursor is denoted as the change in enthalpy Δ*H*. Fig. 11b reveals that the estimated Δ*H* values are *α*-dependent, and that the dependence of Δ*H* on *α* follows analogous form to that of Δ*S* and activation energy. The average values of Δ*H* (52.86, 101.34, 175.0 kJ mol^−1^) for Step 4, -5 and -6 are lower than the average activation energy values (56.92, 105.87 and 179.76 kJ mol^−1^) of the corresponding steps, which indicate the formation of the activated complexes. The small difference between the activation energy and change in enthalpy suggests a lower energy barrier between the products produced and the activated complex.^63^ Hence, there is a smaller required amount of thermal energy for the occurrence of the reactions. The variation of Δ*G* with *α* for the above-stated reactions is shown in Fig. 11c. Δ*G* is the difference in the free energies between the activated complex and the precursor. The sign of Δ*G* points to the spontaneity of the reactions. Thus, Δ*G* is the key factor that directs a chemical reaction. Presently, for Step 4, -5 and -6, the Δ*G* values lie within 128.5 to 128.7 kJ mol^−1^, 144.5 to 140.9 kJ mol^−1^ and 159.6 to 152.6 kJ mol^−1^, respectively. As the Δ*G* values are higher than the Δ*H* values for each step, the energy supplied to the system is partially used for the creation of products. For all of the reaction steps, the positive Δ*G* values specify that the present thermal reactions are non-spontaneous and require energy to continue.

### 3.6 Role of the co-precursor

The thermal decomposition of bare ferrocene carboxaldehyde in oxygen atmosphere is a multi-step process completed at ∼678 K, leaving pure hematite as the residual mass. Conversely, the thermal decomposition of the same compound in nitrogen atmosphere completed at ∼560 K yields a mixture of hematite, cementite and magnetite.^37^ Thus, the significant effect of the oxygen atmosphere on the nature of the thermal decomposition of ferrocene carboxaldehyde and also on the resultant products are established. Herein, a mixture of ferrocene carboxaldehyde and oxalic acid is thermally decomposed in an oxygen atmosphere, and produced hematite at 625 K, as confirmed from the XRD study, which also recognized the effect of co-precursor on the reaction process of ferrocene carboxaldehyde in oxygen atmosphere. Ferrocene carboxaldehyde is a substituted derivative of ferrocene (C_5_H_5_)_2_Fe. It has two parallel cyclopentadienyl rings with an iron ion in the centre between the rings. It is reported that ferrocene sublimates at ∼448 K. Above ∼773 K, gaseous ferrocene decomposes spontaneously, giving rise to metallic iron along with some volatile gases [(C_5_H_5_)_2_Fe → Fe + H_2_ + CH_4_ + C_5_H_6_ + …].^64^ At such high temperatures, solid or liquid-like iron particles exist in the reaction medium. However, when ferrocene was heated at ∼453 K together with oxalic acid, hematite was formed^35^ [2(C_5_H_5_)_2_Fe + (COOH)_2_·2H_2_O → Fe_2_O_3_ (s) + CO_2_(g) + CO(g) + 13H_2_(g) +22C], where oxalic acid first decomposed to CO_2_ and CO and H_2_O. The CO_2_ and CO are deoxidized to oxygen and carbon by metallic iron, and it then reacted with the available oxygen, giving rise to hematite.^35^ Presently, considering the strength of the bonds present in ferrocene carboxaldehyde, it is obvious that the –CH=O bond will break prior to the breakage of the Fe–C bonds, and the resulting metallic iron will react with the oxygen available to give rise to hematite.^37^

There are many predictions on the thermal decomposition of oxalic acid. According to Lapidus *et al.*,^65^ the thermal decomposition of oxalic acid, at temperatures up to 430 K, yielded only CO_2_ and HCOOH. Multiphoton infrared decomposition study of oxalic acid by Yamamoto and Back^66^ pointed out that though two primary processes yielding CO_2_ + HCOOH and CO_2_ + CO + H_2_O may occur, but CO and H_2_O are more vital products than HCOOH considering the energy required. From the *ab initio* calculations over the temperature range of 300–1300 K, Kakumoto *et al.*^67^ considered the decomposition of oxalic acid to occur through several reaction channels as follows:

a (COOH)_2_ → HCOOH + CO_2_

b (COOH)_2_ → CO_2_ + CO + H_2_O

c (COOH)_2_ → 2CO_2_ + H_2_

d (COOH)_2_ → 2COOH

e
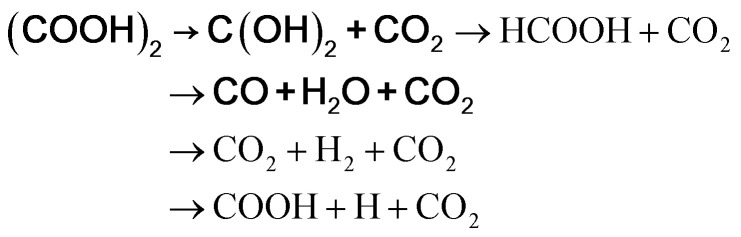
where it was predicted that the lowest energy path was (COOH)_2_ → CO_2_ + CO + H_2_O (in bold), as the dihydroxycarbene [C(OH)_2_] produced on decomposition of oxalic acid in path (e) mostly decomposes to CO + H_2_O. Kakumoto *et al.*^67^ performed thermal decomposition of oxalic acid vapor in an inert atmosphere in 850–1300 K, which led to CO_2_, CO, and H_2_O as the major products. This is consistent with their proposed reaction path obtained from the *ab initio* calculations, and also the infrared multiphoton study by Yamamoto and Back.^66^ According to Higgins *et al.*,^68^ although rapid formation of CO_2_ and HCOOH in gas phase oxalic acid thermal decomposition is possible, with increasing temperature, the production of CO_2_, CO, and H_2_O becomes more significant.

Presently, the 1^st^ peak that appeared for the decomposition of the bare oxalic acid dihydrate arises due to evaporation of absorbed water present, and the dehydrated oxalic acid further decomposes in two-steps. It should be noted that during the thermal decomposition of oxalic acid dihydrate in the presence of ferrocene carboxaldehyde, the third step of oxalic acid thermal decomposition did not appear. Thus, Step 2 of the oxalic acid decomposition process may be recognized as the formation of C(OH)_2_ and CO_2_, following Kakumoto *et al.*^67^ The [C(OH)_2_] produced on further heating decomposes to CO + H_2_O. Notably, thermal decomposition of ferrocene carboxaldehyde is also initiated at around the same temperature. Looking at the complexity of the reaction, it may plausibly be inferred that the metallic iron produced (as discussed above) will react with the oxygen available in the reaction environment to form hematite. Additionally, the metallic iron may deoxidize CO_2_ into carbon and oxygen,^34^ and react with this additional oxygen as an additional reaction, forming hematite. As no traceable carbon or iron carbide is detected in the residual product, it is likely that the carbon produced as an intermediate might have been oxidized to CO_2_/CO.^69^

Table 2 compares the temperature range, activation energy, reaction mechanism functions and reaction rate observed for thermal decomposition of FO_11_ and FcCHO in an O_2_ atmosphere. It is clear from this table that the use of oxalic acid as a co-precursor has significantly reduced the temperature at which the step-wise thermal decomposition reaction occurred in FcCHO, modified the activation energy of the thermal decomposition reaction of FcCHO particularly in Step 5, and enhanced the reaction rates by several orders in Step 5 and -6, but did not meaningfully change the reaction mechanisms in Step 4 and -5. When the changes involved in the thermodynamic parameters are compared, no significant change in them are observed for Step 4, whereas a huge change in those parameters are observed for Step 5 in comparison to those in Step 6. Perhaps in FO_11_, the reactivity of the reactants after Step 3 is higher, which led to the early completion of the reaction for FO_11_ with respect to bare FcCHO on the temperature scale. Thus, the oxalic acid co-precursor acts as a reaction enhancer^70^ in the thermal decomposition of ferrocene carboxaldehyde. It may be recalled that ferrocene sublimates at ∼773 K. When ferrocene is heated together with oxalic acid, it produces hematite at much lower temperature (∼453 K)^35^ as is seen in the present case.

**Table 2:** A comparison of the kinetic parameters (*E*_*α*_, *g*(*α*), and *A*_*α*_) and thermodynamic parameters (Δ*S*, Δ*H*, and Δ*G*) for Step 4, 5, and 6 observed for the thermal decomposition of FO_11_ and ferrocene carboxaldehyde (FcCHO)

Parameters	Material	Step 4	Step 5	Step 6
Steps (K)	FO_11_	439–515	440–568	515–625
	FcCHO^a^	472–512	452–641	585–678
*E*_*α*_ (kJ mol^−1^)	FO_11_	52–67	82–137	128–209
	FcCHO^a^	40–81	45 – 105	165 – 235
*g*(*α*)^b^	FO_11_	[−ln (1 − *α*)]^1/2^, *A*_2_	[−ln (1 − *α*)]^2/3^, *A*_3/2_	[−ln (1 − *α*)]^2/3^, *F*_3/2_
	FcCHO^a^	[−ln (1 − *α*)]^1/3^, *A*_3_	[−ln (1 − *α*)]^2/3^, *A*_3/2_	1 − (1 − *α*)^1/2^, *R*_2_/*F*_1/2_
*A*_*α*_ at *α* = 0.5 (min^−1^)	FO_11_	8.4 × 10^4^	3.8 × 10^9^	2.1 × 10^16^
	FcCHO^a^	2.3 × 10^5^	9.2 × 10^5^	4.5 × 10^13^
Av. Δ*S* (J mol^−1^ K^−1^)	FO_11_	−154.8	−74.4	36.6
	FcCHO^a^	−150.6	−126.8	−15.3
Av. Δ*H* (kJ mol^−1^)	FO_11_	52.9	101.3	175.0
	FcCHO^a^	58.7	83.3	181.6
Av. Δ*G* (kJ mol^−1^)	FO_11_	128.4	141.9	154.1
	FcCHO^a^	137.6	162.6	191.8

a 25.

b For symbols used, refer to Table S1.

## 4. Conclusion

Thermal decomposition of ferrocene carboxaldehyde in the presence of oxalic acid under an oxygen atmosphere was systematically studied with the help of non-isothermal thermogravimetry. The decomposed product was identified to be pure hematite of nano-rod shape. The multistep thermogravimetry profiles were successfully deconvoluted, which showed that the decomposition occurs in six individual steps. It was analysed that the last three reaction steps were responsible for the production of hematite. The reaction kinetics for these three steps of decomposition were studied. Step-wise systematic dependence of the activation energy on the extent of conversion was observed. Here, the thermal decomposition progressed through different reaction mechanisms and with different range of reaction rates. Reactions between the gaseous products formed due to the decomposition of oxalic acid, and the intermediate product obtained on decomposition of ferrocene carboxaldehyde were plausibly held accountable for the production of hematite. Use of a co-precursor significantly enhanced the thermal reaction, lowered the reaction completion temperature, and modified the activation energy and reaction rate. Probably, during such reactions, the reactivity of the reactant particles is modified by factors like defect formation, particle disintegration, development of crystal strain, *etc.* Presently, by increasing the temperature during the decomposition, these factors might have been severely affected, which resulted in the different reaction mechanism functions for each step. This affected the dependence of the activation energy and reaction rate on the extent of conversion for each step. The present study describes the reaction kinetics of a co-precursor-driven solid state reaction, leading to hematite nanomaterial, and proposes that a suitable blend of precursor and co-precursor may produce important iron oxide nanomaterials of various sizes through solid-state thermal decomposition.

## Author contributions

Material preparation, data collection and analysis were performed by M. C., whereas S. K. was involved in some data collection and analysis. A. B. conceptualized the problem and designed the study. The first draft of the manuscript was written by M. C. and finalized by A. B. All authors reviewed the final manuscript.

## Conflicts of interest

The authors declare that they have no conflict interest.

## Supplementary Material

RA-013-D3RA07045J-s001
