# Supplementary material for: Insights into a co-precursor driven solid-state thermal reaction of ferrocene carboxaldehyde leading to hematite nanomaterial: a reaction kinetic study
Source: RSC Adv. 2023 Dec 1;13(50):34972–86. doi: 10.1039/d3ra07045j (PMC10690136; doi:10.1039/d3ra07045j)
Supplement: RA-013-D3RA07045J-s001 [file RA-013-D3RA07045J-s001.pdf]

Electronic Supplementary Material (ESI) for RSC Advances.
This journal is © The Royal Society of Chemistry 2023

Supplementary Information

Insights into a Co-precursor Driven Solid-State Thermal Reaction of Ferrocene carboxaldehyde Leading to Hematite Nanomaterial through Reaction Kinetic Study

Manisha Chakraborty, Sani Kundu, Ashis Bhattacharjee*
Department of Physics, Institute of Science, Visva-Bharati University, Santiniketan, 731235, India

Table S1. The frequently used kinetic models with mechanisms operating in solid-state reactions [52].

Sl.no.	Type of mechanism	Mechanism	f(α)	g(α)	Function name
1	Chemical Process or mechanism non-invoking equations	Chemical Reaction	$(3/2)(1-\alpha)^{2/3}$	$1-(1-\alpha)^{2/3}$	One-third order, $F_{1/3}$
2			$4(1-\alpha)^{3/4}$	$1-(1-\alpha)^{1/4}$	Three- quarters order, $F_{3/4}$
3			$2(1-\alpha)^{3/2}$	$(1-\alpha)^{-1/2}-1$	One and a half order, $F_{3/2}$
4			$(1-\alpha)^{2}$	$(1-\alpha)^{-1}-1$	Second order, F_2
5			$(1/2)(1-\alpha)^{3}$	$(1-\alpha)^{-2}-1$	Third order, F_3
6	Acceleratory rate equations	Nucleation	$(2/3)\alpha^{-1/2}$	$\alpha^{3/2}$	Mampel power law, $P_{3/2}$
7			$2\alpha^{1/2}$	$\alpha^{1/2}$	Mampel power law, $P_{1/2}$
8			$3\alpha^{2/3}$	$\alpha^{1/3}$	Mampel power law, $P_{1/3}$
9			$4\alpha^{1/4}$	$\alpha^{1/4}$	Mampel power law, $P_{1/4}$
10			α	$\ln\alpha$	Exponential law, E_1
11	Sigmoidal rate Equations or Random nucleation and subsequent growth	Assumed random nucleation and its subsequent growth, n=1	$(1-\alpha)$	$-\ln(1-\alpha)$	Avrami-Erofeev equations, A_1, F_1
12		Assumed random nucleation and its subsequent growth, n=1.5	$(3/2)(1-\alpha)[-\ln(1-\alpha)]^{1/3}$	$[-\ln(1-\alpha)]^{2/3}$	Avrami-Erofeev equations, $A_{3/2}$
13		Assumed random nucleation and its subsequent growth, n=2	$2(1-\alpha)[-\ln(1-\alpha)]^{1/2}$	$[-\ln(1-\alpha)]^{1/2}$	Avrami-Erofeev equations, A_2
14		Assumed random nucleation and its subsequent growth, n=3	$3(1-\alpha)[-\ln(1-\alpha)]^{2/3}$	$[-\ln(1-\alpha)]^{1/3}$	Avrami-Erofeev equations, A_3
15		Assumed random nucleation and its subsequent growth, n=4	$4(1-\alpha)[-\ln(1-\alpha)]^{3/4}$	$[-\ln(1-\alpha)]^{1/4}$	Avrami-Erofeev equations, A_4
16		Branching nuclei	$\alpha(1-\alpha)$	$\ln[\alpha/(1-\alpha)]$	Prout-Tomkins equations, Au
17	Deceleratory	Contracting disk	$(1-\alpha)^{0}$	α	Power law, R_1,

	rate				F_0, P_1
18	equations: Phase	Contracting cylinder (cylindrical symmetry)	$2(1-\alpha)^{1/2}$	$1-(1-\alpha)^{1/2}$	Power law, R_2, $F_{1/2}$
19	boundary reaction	Contracting sphere (Spherical symmetry)	$3(1-\alpha)^{2/3}$	$1-(1-\alpha)^{1/3}$	Power law, R_3, $F_{2/3}$
20	Deceleratory rate	1D diffusion	$1/2\alpha$	α^2	Parabola law, D_1
21	equations: Based on the	2D diffusion	$[-\ln(1-\alpha)]^{-1}$	$\alpha+(1-\alpha)\ln(1-\alpha)$	Valensi equation, D_2
22	Diffusion mechanism	3D diffusion, Spherical symmetry	$(3/2)(1-\alpha)^{2/3}[1-(1-\alpha)^{1/3}]^{-1}$	$[1-(1-\alpha)^{1/3}]^2$	Jander equation, D_3
23		3D diffusion, Cylindrical symmetry	$(3/2)[(1-\alpha)^{-1/3}-1]^{-1}$	$1-2\alpha/3-(1-\alpha)^{2/3}$	Ginstling-Brounstein equation, D_4
24		3D diffusion	$(3/2)(1-\alpha)^{4/3}[(1-\alpha)^{-1/3}-1]^{-1}$	$[(1-\alpha)^{-1/3}-1]^2$	Zhuravlev, Lesokin, Tempelman equation, D_5
25		3D diffusion	$(3/2)(1+\alpha)^{4/3}[(1+\alpha)^{-1/3}-1]^{-1}$	$[(1+\alpha)^{1/3}-1]^2$	Anti-Jander equation, D_6
26		3D diffusion	$(3/2)[(1+\alpha)^{-1/3}-1]^{-1}$	$1+2\alpha/3-(1+\alpha)^{2/3}$	Anti-Ginstling-Brounstein equation, D_7
27		3D diffusion	$(3/2)(1+\alpha)^{4/3}[(1+\alpha)^{-1/3}-1]^{-1}$	$[(1+\alpha)^{-1/3}-1]^2$	Anti-Zhuravlev, Lesokin, Tempelman equation, D_8
28	Other	-	$1/2(1-\alpha)$	$1-(1-\alpha)^2$	G_1
29	kinetics	-	$1/3(1-\alpha)^2$	$1-(1-\alpha)^3$	G_2
30	equations	-	$1/4(1-\alpha)^3$	$1-(1-\alpha)^4$	G_3
31	with unjustified	-	$(1/2)(1-\alpha)[-\ln(1-\alpha)]^{-1}$	$[-\ln(1-\alpha)]^2$	G_4
32	mechanism	-	$(1/3)(1-\alpha)[-\ln(1-\alpha)]^{-2}$	$[-\ln(1-\alpha)]^3$	G_5
33		-	$(1/4)(1-\alpha)[-\ln(1-\alpha)]^{-3}$	$[-\ln(1-\alpha)]^4$	G_6
34		-	$4\{(1-\alpha)[1-\ln(1-\alpha)]^{1/2}\}^{1/2}$	$[1-(1-\alpha)^{1/2}]^{1/2}$	G_7
35		-	$6\{(1-\alpha)^{2/3}[1-\ln(1-\alpha)]^{1/3}\}^{1/2}$	$[1-(1-\alpha)^{1/2}]^{1/2}$	G_8

Table S2 Comparison of the fit parameters following Fraser-Suzuki equation for different steps of thermal decomposition of OxA, FcCHO and FO11 in O_2 atmosphere.

FO11 at 3 K min^{-1}

Parameter	Step-1	Step-2	Step-3	Step-4	Step-5	Step-6
h (K^{-1})	0.003 ± 1.03×10^{-5}	0.003 ± 2.35×10^{-5}	0.006 ± 6.87 ×10^{-5}	5.38×10^{-4} ± 1.33×10^{-5}	0.007 ± 9.24×10^{-5}	0.006 ± 6.32×10^{-5}
p (K)	364.39 ± 0.13	408.12 ± 0.19	433.14 ± 0.25	465.07 ± 0.65	530.19 ± 0.40	564.48 ± 0.35
w (K)	41.59 ± 0.20	27.80 ± 0.35	28.65 ± 0.38	30.34 ± 1.53	36.65 ± 0.61	40.67 ± 0.91
s (-)	-0.68 ± 0.01	-0.46 ± 0.03	-0.13 ± 0.03	0.25 ± 0.11	-0.32 ± 0.03	0.15 ± 0.04
r^2	0.99	0.99	0.99	0.97	0.99	0.99
ΔT (K)	300-390	332-432	382-468	439-515	440-568	515-625

FcCHO at 3 K min^{-1}

h (K^{-1})	0.005 ± 1.01×10^{-4}	0.001 ± 8.72×10^{-5}	0.002 ± 8.00×10^{-5}	9.63×10^{-4} ± 5.18×10^{-5}	0.004 ± 3.97×10^{-5}	0.018 ± 9.74×10^{-5}
p (K)	432.19 ± 0	465.53 ± 0	481.69 ± 0	493.14 ± 0	600.67 ± 0.59	642.87 ± 0.13
w (K)	16.16 ± 0	12.13 ± 0	11.02 ± 0	10.34 ± 0	58.46 ± 0.95	29.30 ± 0.45
s (-)	-0.86 ± .0	-0.38 ± 0	-0.15 ± .0	0.25 ± .0	-0.56 ± .04	-0.19 ± .03
r^2	0.97	0.83	0.94	0.94	0.98	0.99
ΔT (K)	380-440	446-479	464-494	472-512	452-641	585-678

OxA at 5 K min^{-1}

h (K^{-1})	0.001 ± 9.52×10^{-5}	4.80×10^{-4} ± 1.78×10^{-5}	0.004 ± 0.001
p (K)	375.38 ± 0.15	402.44 ± 0.95	468.40 ± 0.20
w (K)	19.71 ± 0.28	23.14 ± 1.89	10.30 ± 0.50
s (-)	-0.84 ± 0.02	-0.44 ± 0.24	-1.15 ± 0.09
r^2	0.99	0.80	0.94
ΔT (K)	304-387	378-414	380-474

h = peak amplitude, p = peak temperature, w = half width and s = asymmetry, r^2 = co-relational factor, ΔT = range of step.

Table S3 The fit parameters following Fraser-Suzuki equation for different steps of thermal decomposition of FO11 in O_2 atmosphere under different heating rates (β)

Parameter	Step-1	Step-2	Step-3	Step-4	Step-5	Step-6
			**β = 3 K min^{-1}**			
h (K^{-1})	0.003 ± 1.03×10^{-5}	0.003 ± 2.35×10^{-5}	0.006 ± 6.87 ×10^{-5}	5.38×10^{-4} ± 1.33×10^{-5}	0.007 ± 9.24×10^{-5}	0.006 ± 6.32×10^{-5}
p (K)	364.39 ± 0.13	408.12 ± 0.19	433.14 ± 0.25	465.07 ± 0.65	530.19 ± 0.40	564.48 ± 0.35
w (K)	41.59 ± 0.20	27.80 ± 0.35	28.65 ± 0.38	30.34 ± 1.53	36.65 ± 0.61	40.67 ± 0.91
**s (-)**	-0.68 ± 0.01	-0.46 ± 0.03	-0.13 ± 0.03	0.25 ± 0.11	-0.32 ± 0.03	0.15 ± 0.04
r^2	0.99	0.99	0.99	0.97	0.99	0.99
ΔT (K)	300-390	332-432	382-468	439-515	440-568	515-625
			**β = 6 K min^{-1}**			
h (K^{-1})	0.003 ± 5.21× 10^{-5}	0.003 ± 3.65×10^{-5}	0.008 ± 9.36×10^{-5}	4.14×10^{-4} ± 1.30×10^{-5}	0.008 ± 9.36×10^{-5}	0.005 ± 5.73×10^{-5}
p (K)	372.95 ± 0	417.85 ± 0.31	443.41 ± 0.27	482.58 ± 1.27	538.25 ± 0.35	570.98 ± 0.28
w (K)	37.49 ± 0.74	26.73 ± 0.49	26.30 ± 0.78	37.84 ± 2.43	35.35 ± 0.53	34.08 ± 0.48
**s (-)**	-0.58 ± 0	-0.65 ± 0.05	-0.22 ± 0.27	-0.12 ± 0.20	-0.31 ± 0.03	0.43 ± 0.03
r^2	0.98	0.99	0.99	0.90	0.99	0.99
ΔT (K)	307-398	337-435	400-468	435-523	473-570	544-646
			**β = 9 K min^{-1}**			
h (K^{-1})	0.003 ± 5.97×10^{-5}	0.004 ± 2.94×10^{-5}	0.007 ± 7.84×10^{-5}	2.25×10^{-4} ± 2.24×10^{-6}	0.009 ± 8.96×10^{-5}	0.007 ± 4.81×10^{-5}
p (K)	372.07 ± 0.55	424.16 ± 0.19	449.53 ± 0.21	479.87 ± 0.17	549.34 ± 0.35	577.48 ± 0.17
w (K)	35.79 ± 0.83	25.85 ± 0.27	23.99 ± 0.88	22.13 ± 0.87	34.46 ± 1.53	30.99 ± 0.27
**s (-)**	-0.37 ± .05	-0.51 ± .03	-0.10 ± 0.06	0.64 ± 0.11	-0.37 ± .05	0.38 ± 0.02
r^2	0.98	0.99	0.99	0.99	0.99	0.99
ΔT (K)	300-402	347-445	406-481	463-520	458-585	549-656
			**β = 15 K min^{-1}**			
h (K^{-1})	0.003 ± 5.29×10^{-5}	0.005 ± 2.02×10^{-5}	0.008 ± 3.36×10^{-5}	3.61×10^{-4} ± 7.91×10^{-6}	0.012 ± 0.00	0.011 ± 1.24×10^{-4}
p (K)	374.32 ± 0.61	429.19 ± 0.14	461.54 ± 0.07	525.56 ± 0.07	562.47 ± 0.21	580.07 ± 0.13
w (K)	39.45 ± 0.89	33.75 ± 0.38	19.59 ± 0.22	28.33 ± 1.18	25.79 ± 0.25	15.45 ± 0.19
**s (-)**	-0.15 ± 0.05	-0.25 ± 0.03	-0.11 ± 0.02	-0.61 ± 0.12	-0.36 ± 0.03	0.03 ± 0.02
r^2	0.98	0.99	0.99	0.97	0.99	0.99
ΔT (K)	310-419	367-465	426-488	494-545	503-586	552-614

h = peak amplitude, p = peak temperature, w = half width and s = asymmetry, r^2 = co-relational factor, ΔT = range of step.

Table S4. Results obtained on linear fitting of ln(β/T^n) vs. 1/T curves following FWO, KAS, Tang and Starink methods (eqs (5a-d)) for different steps of thermal decomposition of FO11 in O_2 atmosphere.

Method	α	Slope *a*	Error	Intercept *b*	Regression R^2
			**Step-4**		
Eq 5a	0.10	-8708.45	1718.98	20.54	0.93
	0.15	-8191.15	1619.37	19.29	0.93
	0.20	-7781.96	1625.39	18.32	0.92
	0.25	-7590.17	1665.56	17.83	0.91
	0.30	-7460.94	1710.80	17.49	0.90
	0.35	-7361.54	1767.89	17.22	0.90
	0.40	-7302.59	1774.48	17.04	0.89
	0.45	-7289.29	1803.04	16.95	0.89
	0.50	-7287.12	1817.94	16.89	0.89
	0.55	-7337.02	1854.47	16.93	0.89
	0.60	-7377.25	1843.41	16.96	0.89
	0.65	-7479.30	1854.96	17.11	0.89
	0.70	-7619.03	1816.24	17.33	0.90
	0.75	-7817.15	1802.79	17.65	0.90
	0.80	-8060.87	1726.07	18.06	0.92
	0.85	-8480.69	1615.15	18.79	0.93
	0.90	-8986.46	1322.96	19.66	0.97
Eq 5b	0.10	-7767.10	1723.35	6.23	0.91
	0.15	-7239.26	1626.74	4.96	0.91
	0.20	-6822.32	1634.25	3.97	0.90
	0.25	-6624.30	1675.02	3.47	0.89
	0.30	-6489.81	1720.56	3.12	0.88
	0.35	-6385.68	1777.71	2.84	0.87
	0.40	-6322.58	1784.42	2.65	0.86
	0.45	-6305.14	1812.96	2.55	0.86
	0.50	-6299.33	1827.86	2.48	0.85
	0.55	-6345.52	1864.28	2.52	0.85
	0.60	-6382.00	1853.29	2.53	0.86
	0.65	-6480.65	1864.79	2.68	0.86
	0.70	-6616.64	1826.16	2.89	0.87
	0.75	-6810.60	1812.76	3.21	0.88
	0.80	-7050.27	1736.13	3.60	0.89
	0.85	-7465.18	1625.32	4.33	0.91
	0.90	-7968.83	1333.02	5.19	0.95
Eq 5c	0.10	-7816.68	1723.11	6.99	0.91
	0.15	-7289.40	1626.35	5.72	0.91
	0.20	-6872.86	1633.78	4.73	0.90
	0.25	-6675.17	1674.52	4.23	0.89
	0.30	-6540.96	1720.05	3.87	0.88
	0.35	-6437.08	1777.19	3.59	0.87
	0.40	-6374.20	1783.89	3.40	0.86
	0.45	-6356.97	1812.44	3.31	0.86
	0.50	-6351.35	1827.34	3.24	0.86
	0.55	-6397.74	1863.77	3.28	0.85
	0.60	-6434.42	1852.77	3.29	0.86
	0.65	-6533.25	1864.27	3.44	0.86
	0.70	-6669.43	1825.63	3.65	0.87
	0.75	-6863.62	1812.23	3.97	0.88
	0.80	-7103.49	1735.60	4.37	0.89
	0.85	-7518.67	1624.78	5.09	0.91
	0.90	-8022.61	1332.49	5.95	0.92

Eq 5d	0.10	-7790.63	1723.24	6.59	0.91
	0.15	-7263.06	1626.56	5.32	0.91
	0.20	-6846.31	1634.02	4.33	0.90
	0.25	-6648.44	1674.79	3.83	0.89
	0.30	-6514.08	1720.32	3.48	0.88
	0.35	-6410.07	1777.47	3.20	0.87
	0.40	-6347.08	1784.17	3.00	0.86
	0.45	-6329.74	1812.72	2.91	0.86
	0.50	-6324.02	1827.61	2.84	0.86
	0.55	-6370.31	1864.04	2.88	0.85
	0.60	-6406.88	1853.05	2.90	0.86
	0.65	-6505.62	1864.54	3.04	0.86
	0.70	-6641.70	1825.91	3.25	0.87
	0.75	-6835.76	1812.50	3.57	0.88
	0.80	-7075.53	1735.87	3.97	0.89
	0.85	-7490.57	1625.07	4.69	0.91
	0.90	-7994.35	1332.77	5.55	0.95
			**Step-5**		
Eq 5a	0.10	-10454.66	1896.79	22.18	0.94
	0.15	-11059.07	1880.13	23.13	0.95
	0.20	-11535.43	1881.06	23.88	0.95
	0.25	-11938.34	1883.59	24.51	0.95
	0.30	-12333.15	1909.56	25.14	0.95
	0.35	-12699.29	1850.69	25.71	0.96
	0.40	-13055.23	1903.28	26.28	0.97
	0.45	-13426.98	1883.28	26.88	0.96
	0.50	-13698.30	1893.32	27.28	0.96
	0.55	-14098.60	1877.43	27.94	0.97
	0.60	-14386.75	1901.29	28.38	0.97
	0.65	-14721.95	1878.83	28.90	0.97
	0.70	-15222.27	1931.69	29.72	0.97
	0.75	-15654.57	1956.58	30.41	0.97
	0.80	-16020.55	1940.11	30.97	0.97
	0.85	-16623.88	1998.63	31.94	0.97
	0.90	-17247.14	1963.08	32.90	0.97
Eq 5b	0.10	-9414.37	1905.03	7.67	0.92
	0.15	-10009.09	1888.00	8.61	0.93
	0.20	-10478.10	1888.62	9.34	0.93
	0.25	-10875.17	1890.86	9.96	0.94
	0.30	-11264.75	1916.58	10.57	0.95
	0.35	-11626.63	1857.45	11.14	0.95
	0.40	-11978.17	1909.89	11.70	0.95
	0.45	-12346.21	1889.67	12.29	0.96
	0.50	-12613.68	1899.44	12.69	0.96
	0.55	-13010.65	1883.41	13.34	0.96
	0.60	-13295.25	1906.95	13.77	0.96
	0.65	-13626.97	1884.28	14.29	0.96
	0.70	-14123.69	1936.90	15.10	0.96
	0.75	-14552.33	1961.49	15.78	0.96
	0.80	-14914.43	1944.78	16.33	0.97
	0.85	-15513.52	2003.01	17.29	0.97
	0.90	-16131.57	1966.87	18.25	0.97
Eq 5c	0.10	-9469.16	1904.59	8.43	0.93
	0.15	-10064.40	1887.59	9.37	0.93
	0.20	-10533.79	1888.22	10.10	0.94
	0.25	-10931.17	1890.48	10.72	0.94
	0.30	-11321.02	1916.21	11.34	0.95
	0.35	-11683.12	1857.09	11.91	0.95

	0.40	-12034.90	1909.54	12.47	0.95
	0.45	-12403.14	1889.34	13.06	0.96
	0.50	-12670.81	1899.12	13.46	0.96
	0.55	-13067.96	1883.09	14.11	0.96
	0.60	-13352.74	1906.66	14.54	0.96
	0.65	-13684.64	1883.99	15.06	0.96
	0.70	-14181.55	1936.63	15.87	0.96
	0.75	-14610.38	1961.23	16.55	0.97
	0.80	-14972.69	1944.53	17.10	0.97
	0.85	-15572.01	2002.78	18.07	0.97
	0.90	-16190.33	1966.67	19.02	0.97
Eq 5d	0.10	-9440.38	1904.83	8.03	0.94
	0.15	-10035.34	1887.80	8.97	0.93
	0.20	-10504.53	1888.43	9.70	0.94
	0.25	-10901.75	1890.68	10.32	0.94
	0.30	-11291.46	1916.40	10.94	0.95
	0.35	-11653.45	1857.28	11.51	0.95
	0.40	-12005.10	1909.73	12.07	0.95
	0.45	-12373.23	1889.51	12.66	0.96
	0.50	-12640.80	1899.29	13.06	0.96
	0.55	-13037.85	1883.26	13.70	0.96
	0.60	-13322.54	1906.81	14.14	0.96
	0.65	-13654.34	1884.14	14.65	0.96
	0.70	-14151.16	1936.77	15.47	0.96
	0.75	-14579.88	1961.37	16.15	0.97
	0.80	-14942.08	1944.66	16.70	0.97
	0.85	-15541.28	2002.90	17.66	0.97
	0.90	-16159.46	1966.77	18.61	0.97
			**Step-6**		
Eq 5a	0.10	-16221.06	747.75	30.81	0.99
	0.15	-17587.17	537.00	33.09	0.99
	0.20	-18572.60	760.98	34.70	0.99
	0.25	-19524.50	455.84	36.25	0.99
	0.30	-20405.88	471.02	37.68	0.99
	0.35	-21207.22	296.80	38.96	0.99
	0.40	-22147.94	578.12	40.49	0.99
	0.45	-22938.33	703.53	41.72	0.99
	0.50	-23770.31	844.41	43.03	0.99
	0.55	-24655.21	1003.15	44.42	0.99
	0.60	-25040.23	1736.02	44.92	0.99
	0.65	-25238.21	1749.16	45.09	0.99
	0.70	-26081.70	2441.41	46.34	0.99
	0.75	-25983.94	3345.21	45.96	0.98
	0.80	-26353.44	4201.49	46.33	0.98
	0.85	-26202.80	5918.69	45.78	0.95
	0.90	-24673.49	7275.57	42.82	0.92
Eq 5b	0.10	-15110.11	742.65	16.17	0.99
	0.15	-16469.73	532.10	18.44	0.99
	0.20	-17450.21	756.39	20.04	0.99
	0.25	-18397.21	451.29	21.58	0.99
	0.30	-19274.26	466.63	23.00	0.99
	0.35	-20071.48	292.48	24.28	0.99
	0.40	-21008.37	574.05	25.79	0.99
	0.45	-21794.64	699.60	27.03	0.99
	0.50	-22622.59	840.62	28.33	0.99
	0.55	-23503.37	999.51	29.70	0.99
	0.60	-23884.20	1732.62	30.20	0.99
	0.65	-24077.62	1745.77	30.36	0.99

	0.70	-24916.19	2438.30	31.61	0.99
	0.75	-24813.00	3342.37	31.21	0.98
	0.80	-25175.96	4198.96	31.57	0.97
	0.85	-25017.81	5916.84	31.01	0.95
	0.90	-23477.93	7274.42	28.04	0.91
Eq 5c	0.10	-15168.62	742.92	16.94	0.99
	0.15	-16528.59	532.35	19.21	0.99
	0.20	-17509.33	756.63	20.81	0.99
	0.25	-18456.58	451.53	22.36	0.99
	0.30	-19333.86	466.86	23.78	0.99
	0.35	-20131.30	292.71	25.05	0.99
	0.40	-21068.39	574.26	26.57	0.99
	0.45	-21854.88	699.81	27.80	0.99
	0.50	-22683.04	840.83	29.10	0.99
	0.55	-23564.04	999.70	30.48	0.99
	0.60	-23945.09	1732.80	30.97	0.99
	0.65	-24138.75	1745.95	31.14	0.99
	0.70	-24977.58	2438.46	32.38	0.99
	0.75	-24874.67	3342.52	31.99	0.98
	0.80	-25237.98	4199.09	32.35	0.97
	0.85	-25080.22	5916.95	31.79	0.95
	0.90	-23540.90	7274.48	28.82	0.91
Eq 5d	0.10	-15137.88	742.78	16.53	0.99
	0.15	-16497.67	532.22	18.80	0.99
	0.20	-17478.27	756.50	20.41	0.99
	0.25	-18425.39	451.40	21.95	0.99
	0.30	-19302.55	466.74	23.37	0.99
	0.35	-20099.87	292.59	24.65	0.99
	0.40	-21036.86	574.15	26.16	0.99
	0.45	-21823.23	699.70	27.39	0.99
	0.50	-22651.29	840.72	28.69	0.99
	0.55	-23532.17	999.60	30.07	0.99
	0.60	-23913.10	1732.71	30.56	0.99
	0.65	-24106.64	1745.85	30.73	0.99
	0.70	-24945.33	2438.38	31.98	0.99
	0.75	-24842.27	3342.44	31.58	0.98
	0.80	-25205.40	4199.02	31.94	0.97
	0.85	-25047.43	5916.89	31.38	0.95
	0.90	-23507.81	7274.45	28.40	0.91

Table S5. Values of the activation energy for Step-4, Step-5 and Step-6 of thermal decomposition of FO11 using eqs 5a-f.

α	E_α (kJ mole^{-1})					
	**Eq 5a**	**Eq 5b**	**Eq 5c**	**Eq 5d**	**Eq 5e**	**Eq 5f**
			**Step-4**			
0.10	68.85 ± 13.60	64.58 ± 14.33	64.89 ± 14.31	64.77 ± 14.33	64.96	64.97
0.15	64.76 ± 12.81	60.19 ± 13.52	60.52 ± 13.50	60.39 ± 13.52	60.6	60.63
0.20	61.52 ± 12.86	56.72 ± 13.59	57.06 ± 13.56	56.92 ± 13.59	57.16	57.20
0.25	60.01 ± 13.17	55.07 ± 13.93	55.42 ± 13.90	55.28 ±13.92	55.53	55.57
0.30	58.99 ± 13.53	53.96 ± 14.30	54.30 ± 14.28	54.16 ± 14.30	54.43	54.46
0.35	58.20 ± 13.98	53.09 ± 14.78	53.44 ± 14.75	53.29 ± 14.78	53.57	53.61
0.40	57.73 ± 14.03	52.57 ± 14.84	52.92 ± 14.81	52.77 ± 14.83	53.06	53.09
0.45	57.63 ± 14.26	52.42 ± 15.07	52.78 ± 15.05	52.63 ± 15.07	52.92	52.95
0.50	57.61 ± 14.38	52.37 ± 15.20	52.73 ± 15.17	52.58 ± 15.19	52.87	52.90
0.55	58.01 ± 14.67	52.76 ± 15.50	53.11 ± 15.47	52.96 ± 15.50	53.26	53.28
0.60	58.32 ± 14.58	53.06 ± 15.41	53.42 ± 15.38	53.27 ± 15.41	53.56	53.59
0.65	59.13 ± 14.67	53.88 ± 15.50	54.24 ± 15.48	54.09 ± 15.50	54.38	54.40
0.70	60.24 ± 14.36	55.01 ± 15.18	55.37 ± 15.16	55.22 ±15.18	55.5	55.52
0.75	61.80 ± 14.26	56.62 ± 15.07	56.98 ± 15.05	56.83 ± 15.07	57.11	57.13
0.80	63.73 ± 13.65	58.62 ± 14.43	58.97 ± 14.41	58.83 ± 14.43	59.09	59.11
0.85	67.05 ± 12.77	62.07 ± 13.51	62.42 ± 13.49	62.28 ± 13.51	62.52	62.54
0.90	71.05 ± 10.46	66.25 ± 11.08	66.60 ± 11.06	66.47 ± 11.08	66.69	66.70
			**Step-5**			
0.10	82.66 ± 15.00	78.27 ± 15.84	78.61 ± 15.82	78.49 ± 15.84	78.66	78.70
0.15	87.43 ± 14.87	83.26 ± 15.70	83.55 ± 15.67	83.43 ± 15.70	83.60	83.62
0.20	91.20 ± 14.88	87.11 ± 15.70	87.45 ± 15.68	87.33 ± 15.70	87.49	87.51
0.25	94.39 ± 14.88	90.42 ± 15.72	90.75 ± 15.69	90.64 ± 15.72	90.78	90.80
0.30	97.51 ± 15.10	93.66 ± 15.93	93.99 ± 15.91	93.88 ± 15.94	94.01	94.03
0.35	100.40 ± 14.64	96.66 ± 15.44	96.99 ± 15.42	96.89 ± 15.44	97.01	97.03
0.40	103.22 ± 15.05	99.59 ± 15.88	99.91 ±15.85	99.81 ± 15.88	99.93	99.94
0.45	106.15 ± 14.90	102.65 ± 15.71	102.97 ± 15.66	102.87 ± 15.71	102.98	103.00
0.50	108.30 ± 14.97	104.87 ± 15.80	105.19 ± 15.77	105.10 ± 15.79	105.20	105.21
0.55	111.46 ± 14.85	108.17 ± 15.66	108.49 ± 15.63	108.40 ± 15.66	108.50	108.51
0.60	113.74 ± 15.04	110.54 ± 15.85	110.85 ± 15.83	110.76 ± 15.85	110.86	110.87

0.65	116.39 ± 14.86	113.29 ± 15.67	113.61 ± 15.64	113.52 ± 15.66	113.61	113.62
0.70	120.35 ± 15.28	117.42 ± 16.10	117.73 ± 16.08	117.65 ± 16.10	117.73	117.74
0.75	123.77 ± 15.47	120.99 ± 16.31	121.29 ± 16.28	121.22 ± 16.31	121.29	121.30
0.80	126.66 ± 15.34	124.00 ± 16.17	124.30 ± 16.14	124.23 ± 16.17	124.30	124.30
0.85	131.43 ± 15.81	128.98 ± 16.65	129.28 ± 16.63	129.21 ± 16.65	129.27	129.28
0.90	136.36 ± 15.52	134.12 ± 16.35	134.41 ± 16.33	134.35 ± 16.35	134.4	134.40
			**Step-6**			
0.10	128.24 ± 5.91	125.63 ± 6.17	125.93 ± 6.17	125.86 ± 6.18	125.92	125.93
0.15	139.05 ± 4.25	136.93 ± 4.42	137.22 ± 4.42	137.16 ± 4.42	137.21	137.21
0.20	146.84 ± 6.02	145.08 ± 6.29	145.36 ± 6.28	145.31 ± 6.29	145.35	145.35
0.25	154.36 ± 3.61	152.95 ± 3.75	153.23 ± 3.75	153.19 ± 3.75	153.21	153.22
0.30	161.33 ± 3.73	160.25 ± 3.88	160.51 ± 3.88	160.48 ± 3.88	160.49	160.50
0.35	167.67 ± 2.35	166.87 ± 2.43	167.13 ± 2.43	167.11 ± 2.43	167.12	167.12
0.40	175.10 ± 4.57	174.66 ± 4.77	174.91 ± 4.77	174.90 ± 4.77	174.9	174.90
0.45	181.35 ± 5.56	181.20 ± 5.82	181.44 ± 5.81	181.44 ± 5.82	181.43	181.43
0.50	187.93 ± 6.68	188.08 ± 6.99	188.31 ± 6.98	188.32 ± 6.99	188.31	188.31
0.55	194.93 ± 7.93	195.41 ± 8.31	195.63 ± 8.30	195.65 ± 8.31	195.62	195.63
0.60	197.97 ± 13.73	198.57 ± 14.41	198.80 ± 14.39	198.81 ± 14.41	198.79	198.79
0.65	199.53 ± 13.83	200.18 ± 14.51	200.40 ± 14.49	200.42 ± 14.52	200.39	200.40
0.70	206.20 ± 19.31	207.15 ± 20.27	207.36 ± 20.24	207.40 ± 20.27	207.36	207.37
0.75	205.43 ± 26.46	206.30 ± 27.79	206.51 ± 27.75	206.54 ± 27.79	206.51	206.51
0.80	208.35 ± 33.23	209.31 ± 34.91	209.52 ± 34.86	209.56 ± 34.91	209.52	209.53
0.85	207.16 ± 46.81	208.00 ± 49.19	208.22 ± 49.12	208.24 ± 49.19	208.21	208.22
0.90	195.07 ± 57.54	195.20 ± 60.48	195.44 ± 60.39	195.44 ± 60.48	195.43	195.43

Table S6 Equations used for fitting the E_α vs. α data.

Steps	Equation	R^2
Step-4	$\ln A_\alpha = 6.76 - 8.02\ \alpha + 8.41\ \alpha^2$	0.97
Step-5	$\ln A_\alpha = 6.34 + 6.55\ \alpha - 0.02\ \alpha^2$	0.99
Step-6	$\ln A_\alpha = 7.44 + 26.02\ \alpha - 16.23\ \alpha^2$	0.99

Table S7. Values of Σδ for 35 mechanism functions for Step-4, Step-5 and Step-6 of thermal decomposition of FO11.

Sl. No.	Mechanism			Σδ		
	Type	Symbol	g(α)	Step-4	Step-5	Step-6
1	Chemical	$F_{1/3}$	$1-(1-\alpha)^{2/3}$	0.27433	0.14638	1.16998
2	Process	$F_{3/4}$	$1-(1-\alpha)^{1/4}$	0.47380	0.31192	0.91623
3	or mechanism	$F_{3/2}$	$(1-\alpha)^{-1/2}-1$	1.31702	1.1686	**0.25947**
4	non-invoking	F_2	$(1-\alpha)^{-1}-1$	2.55671	2.41847	1.41779
5	equations	F_3	$(1-\alpha)^{-2}-1$	9.98395	9.86313	9.32872
6		$P_{3/2}$	$\alpha^{3/2}$	0.43936	0.27573	1.03020
7	Acceleratory	$P_{1/2}$	$\alpha^{1/2}$	0.22601	0.37275	1.54081
8	rate	$P_{1/3}$	$\alpha^{1/3}$	0.28697	0.45265	1.61313
9	equations	$P_{1/4}$	$\alpha^{1/4}$	0.32354	0.49517	1.64930
10		E_1	$\ln \alpha$	1.49909	1.67327	2.62755
11	Sigmoidal rate	A_1, F_1	$-\ln(1-\alpha)$	0.66987	0.51084	0.70173
12	Equations or	$A_{3/2}$	$[-\ln(1-\alpha)]^{2/3}$	0.23598	**0.12068**	1.14844
13	Random	A_2	$[-\ln(1-\alpha)]^{1/2}$	**0.11466**	0.18829	1.32912
14	nucleation	A_3	$[-\ln(1-\alpha)]^{1/3}$	0.17618	0.33614	1.48725
15	and subsequent	A_4	$[-\ln(1-\alpha)]^{1/4}$	0.24094	0.41115	1.55979
16	growth	A_u	$\ln[\alpha/(1-\alpha)]$	NaN	NaN	NaN
17		R_1, F_0, P_1	α	0.20954	0.17827	1.30775
18		R_2, $F_{1/2}$	$1-(1-\alpha)^{1/2}$	0.33711	0.18398	1.08148
19		R_3, $F_{2/3}$	$1-(1-\alpha)^{1/3}$	0.42221	0.26106	0.97621
20		D_1	α^2	0.79086	0.62247	0.71120
21	Deceleratory	D_2	$\alpha+(1-\alpha)\ln(1-\alpha)$	1.20783	1.04608	0.44501
22	rate	D_3	$[1-(1-\alpha)^{1/3}]^2$	2.04676	1.89467	0.91758
23	equations	D_4	$1-2\alpha/3-(1-\alpha)^{2/3}$	1.44433	1.28589	0.45918
24		D_5	$[(1-\alpha)^{-1/3}-1]^2$	6.29401	6.15985	5.37532
25		D_6	$[(1+\alpha)^{1/3}-1]^2$	0.58080	0.41134	0.90520
26		D_7	$1+2\alpha/3-(1+\alpha)^{2/3}$	0.64246	0.47276	0.84452
27		D_8	$[(1+\alpha)^{-1/3}-1]^2$	0.43028	0.26991	1.06951
28		G_1	$1-(1-\alpha)^2$	0.2572	0.35959	1.53452
29	Other kinetics	G_2	$1-(1-\alpha)^3$	0.32085	0.45253	1.63156
30	equations with	G_3	$1-(1-\alpha)^4$	0.35537	0.50110	1.67816
31	unjustified	G_4	$[-\ln(1-\alpha)]^2$	3.49366	3.35055	2.40372
32	mechanism	G_5	$[-\ln(1-\alpha)]^3$	12.09433	11.96375	11.53107
33		G_6	$[-\ln(1-\alpha)]^4$	39.15807	39.03711	40.26164
34		G_7	$[1-(1-\alpha)^{1/2}]^{1/2}$	0.16134	0.29391	1.45539
35		G_8	$[1-(1-\alpha)^{1/2}]^{1/2}$	0.13903	0.26146	1.41865

NaN= not a number.

Table S8. Values of A_α of thermal decomposition of FO11 for Step-4, Step-5 and Step-6.

α	A_α (min^{-1})		
	Step-4	Step-5	Step-6
0.10	1.62×10^{6}	5.60×10^{6}	9.88×10^{9}
0.15	5.35×10^{5}	1.99×10^{7}	1.61×10^{11}
0.20	2.24×10^{5}	5.33×10^{7}	1.18×10^{12}
0.25	1.51×10^{5}	1.21×10^{8}	7.60×10^{12}
0.30	1.16×10^{5}	2.66×10^{8}	4.12×10^{13}
0.35	9.56×10^{4}	5.50×10^{8}	1.89×10^{14}
0.40	8.53×10^{4}	1.10×10^{9}	1.08×10^{15}
0.45	8.37×10^{4}	2.27×10^{9}	4.60×10^{15}
0.50	8.43×10^{4}	3.80×10^{9}	2.08×10^{16}
0.55	9.46×10^{4}	8.19×10^{9}	1.01×10^{17}
0.60	1.03×10^{5}	1.41×10^{10}	1.99×10^{17}
0.65	1.29×10^{5}	2.64×10^{10}	2.80×10^{17}
0.70	1.73×10^{5}	6.73×10^{10}	1.21×10^{18}
0.75	2.61×10^{5}	1.50×10^{11}	9.80×10^{17}
0.80	4.30×10^{5}	2.93×10^{11}	1.77×10^{18}
0.85	1.01×10^{6}	8.89×10^{11}	1.28×10^{18}

Table S9 Values of thermodynamic parameters of thermal decomposition of FO11 for Step-4, Step-5 and Step-6.

α	Δ*S* (J mole^{-1} K^{-1})			Δ*H* (kJ mole^{-1})			Δ*G* (kJ mole^{-1})		
	Step-4	Step-5	Step-6	Step-4	Step-5	Step-6	Step-4	Step-5	Step-6
0.10	-138.47	-129.14	-67.28	60.91	74.17	121.18	128.49	144.53	159.59
0.15	-147.68	-118.55	-44.06	56.57	79.09	132.46	128.64	143.68	157.62
0.20	-154.91	-110.38	-27.53	53.14	82.98	140.60	128.75	143.12	156.32
0.25	-158.22	-103.56	-12.04	51.51	86.27	148.47	128.73	142.69	155.34
0.30	-160.40	-96.99	2.03	50.40	89.50	155.75	128.69	142.35	154.60
0.35	-162.04	-90.97	14.68	49.55	92.50	162.37	128.63	142.07	153.99
0.40	-162.99	-85.20	29.20	49.03	95.41	170.15	128.58	141.83	153.49
0.45	-163.15	-79.19	41.22	48.89	98.47	176.68	128.52	141.61	153.15
0.50	-163.10	-74.90	53.76	48.84	100.68	183.56	128.44	141.49	152.87
0.55	-162.15	-68.50	66.93	49.22	103.98	190.88	128.36	141.30	152.67
0.60	-161.40	-63.98	72.55	49.53	106.34	194.04	128.30	141.20	152.63
0.65	-159.54	-58.77	75.40	50.34	109.09	195.6	128.21	141.11	152.61
0.70	-157.12	-50.98	87.53	51.46	113.21	202.62	128.14	140.99	152.66
0.75	-153.68	-44.32	85.79	53.07	116.77	201.76	128.08	140.92	152.78
0.80	-149.51	-38.76	90.68	55.05	119.77	204.78	128.02	140.89	153.0
0.85	-142.42	-29.52	87.99	58.48	124.75	203.47	127.99	140.83	153.24
0.90	-134.04	-20.25	65.37	62.64	129.87	190.68	128.07	140.90	153.36

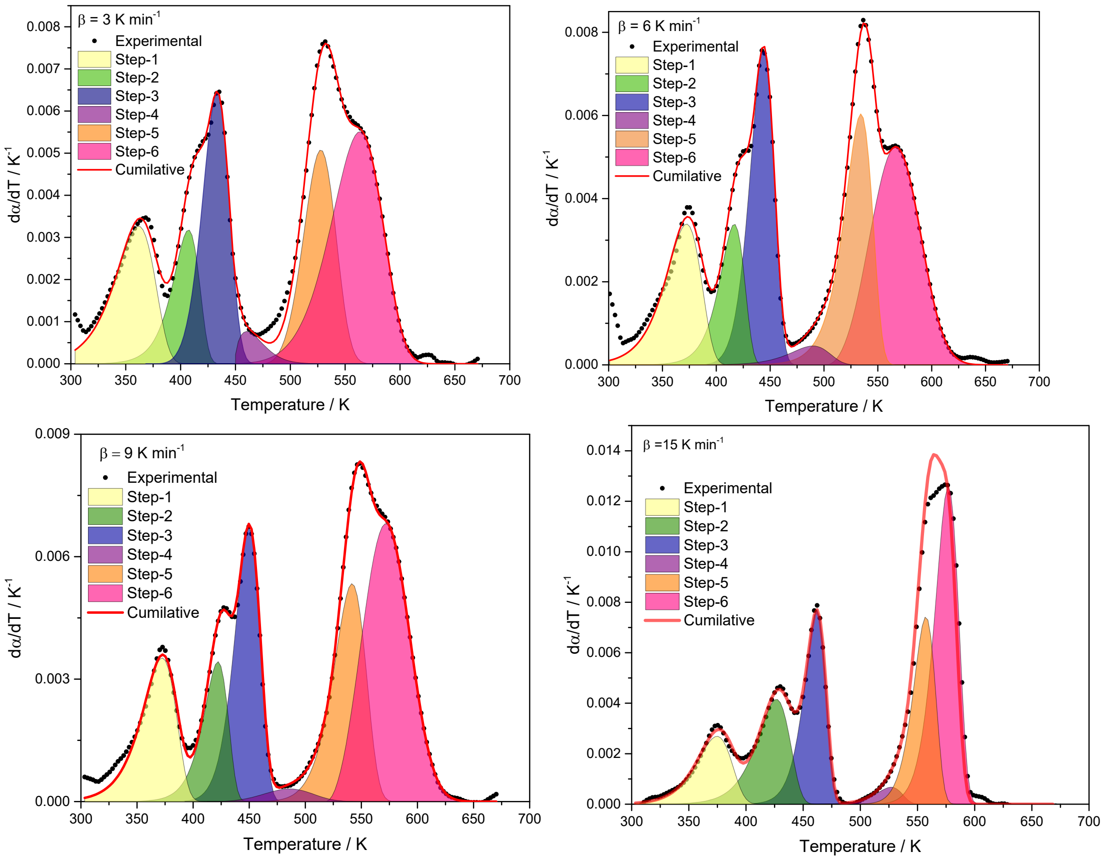

Figure S1 $\frac{d\alpha}{dT}$ **vs.** T **plots for thermal decomposition of FO11 following Weibull function**

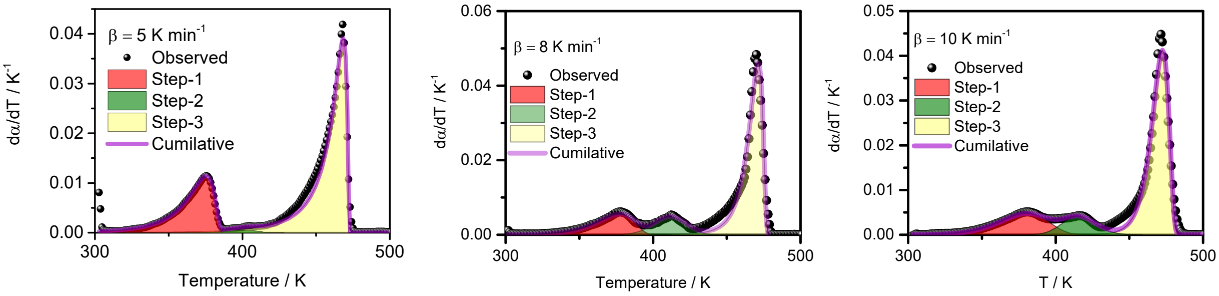

Figure S2 $\frac{d\alpha}{dT}$ **vs.** T **plots for OxA following Fraser Suzuki function**

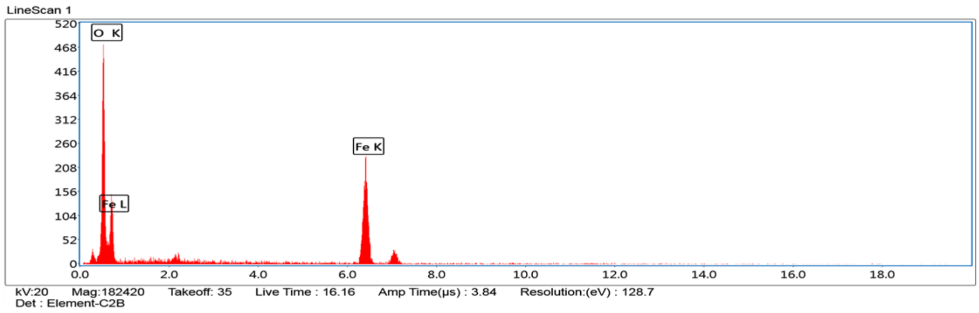

Figure S3 EDX spectrum of the decomposed material obtained from FO11.

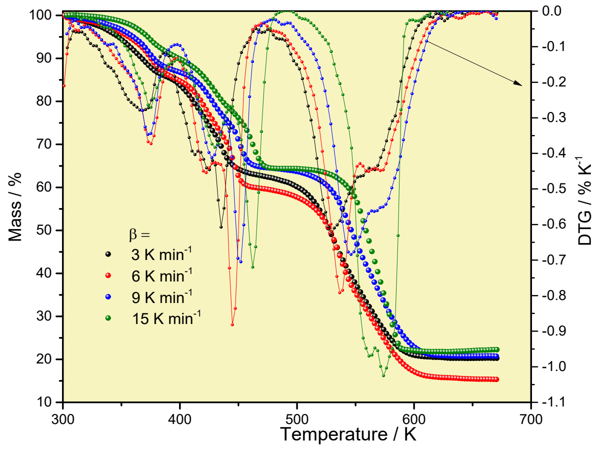

Figure S4 TG and DTG profiles of FO11 for four different heating rates. Arrow indicates the scale used.

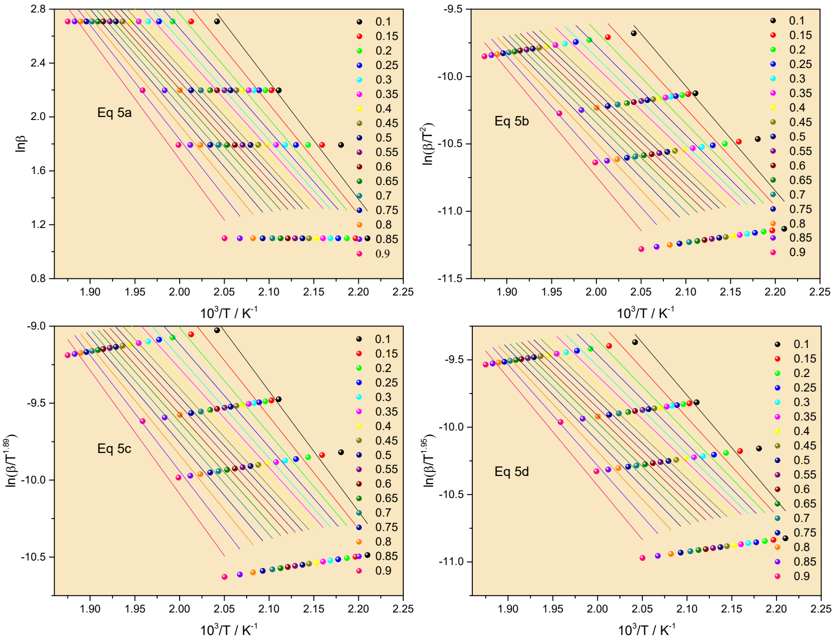

Figure S5-1 The ln(β/T^n) vs. 1/T curves obtained for Step-4 of thermal decomposition of FO11 for different kinetic methods.

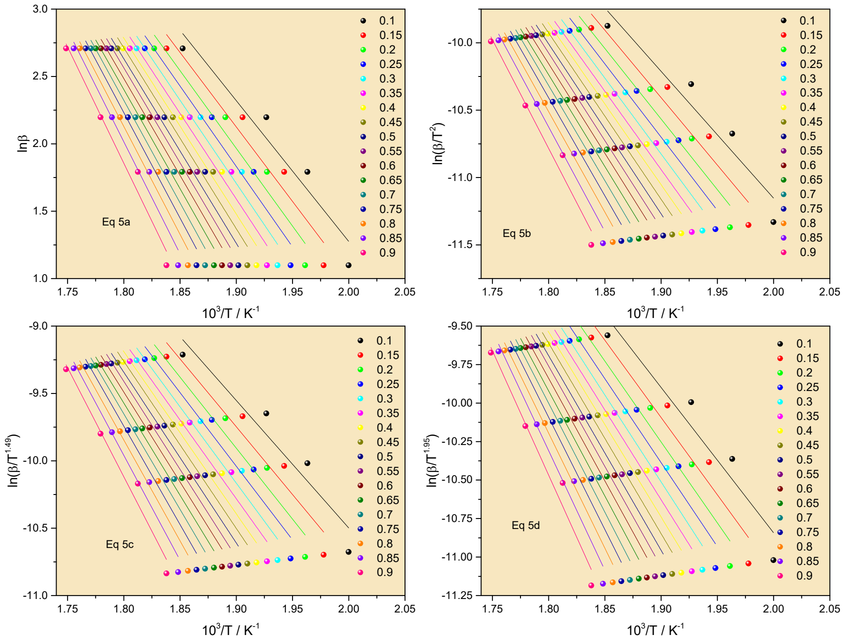

Figure S5-2 The ln(β/T^n) vs. 1/T curves obtained for Step-5 of thermal decomposition of FO11 for different kinetic methods.

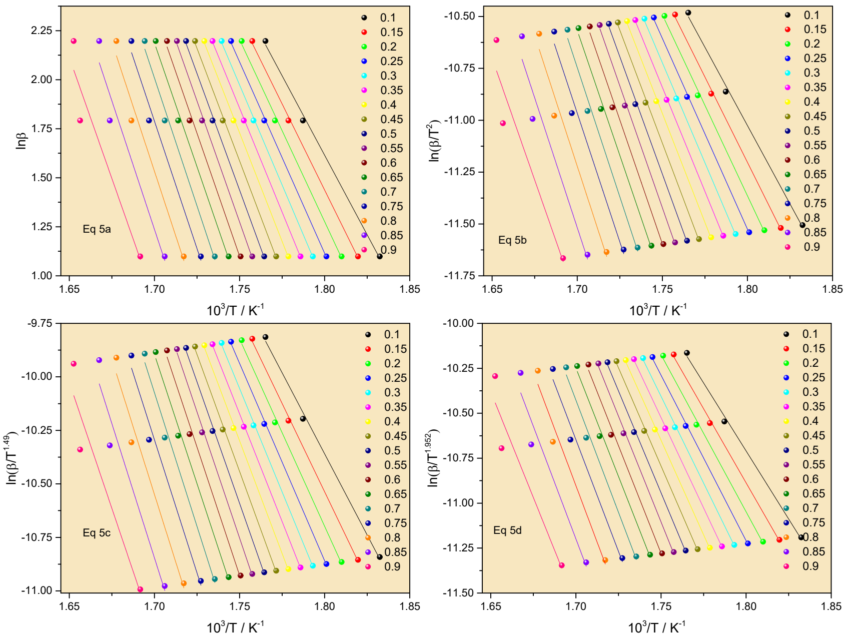

Figure S5-3 The ln(β/T^n) vs. 1/T curves obtained for Step-6 of thermal decomposition of FO11 for different kinetic methods.

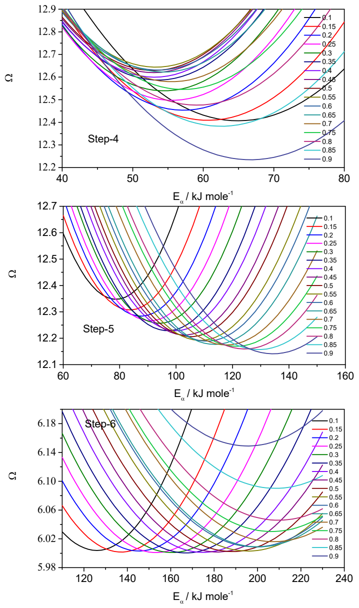

Figure S6 Ω vs. E_α curves for Step-4, Step-5 and Step-6 of FO11.
